# ALS Clinical Trials Review: 20 Years of Failure. Are We Any Closer to Registering a New Treatment?

**DOI:** 10.3389/fnagi.2017.00068

**Published:** 2017-03-22

**Authors:** Dmitry Petrov, Colin Mansfield, Alain Moussy, Olivier Hermine

**Affiliations:** ^1^AB ScienceParis, France; ^2^Imagine Institute, Necker HospitalParis, France; ^3^INSERM, Laboratory of Cellular and Molecular Mechanisms of Hematological Disorders and Therapeutic Implications, UMR 1163Paris, France; ^4^Imagine Institute, Paris Descartes–Sorbonne Paris Cité UniversityParis, France; ^5^CNRS, ERL 8254Paris, France; ^6^Laboratory of Excellence GR-ExParis, France; ^7^Equipe Labélisée par la Ligue Nationale Contre le CancerParis, France; ^8^Department of Hematology, Necker HospitalParis, France

**Keywords:** amyotrophic lateral sclerosis (ALS), motor neuron disease, edaravone, masitinib, clinical trial, riluzole

## Abstract

Amyotrophic lateral sclerosis (ALS) is a devastating condition with an estimated mortality of 30,000 patients a year worldwide. The median reported survival time since onset ranges from 24 to 48 months. Riluzole is the only currently approved mildly efficacious treatment. Riluzole received marketing authorization in 1995 in the USA and in 1996 in Europe. In the years that followed, over 60 molecules have been investigated as a possible treatment for ALS. Despite significant research efforts, the overwhelming majority of human clinical trials (CTs) have failed to demonstrate clinical efficacy. In the past year, oral masitinib and intravenous edaravone have emerged as promising new therapeutics with claimed efficacy in CTs in ALS patients. Given their advanced phase of clinical development one may consider these drugs as the most likely near-term additions to the therapeutic arsenal available for patients with ALS. In terms of patient inclusion, CT with masitinib recruited a wider, more representative, less restrictive patient population in comparison to the only successful edaravone CT (edaravone eligibility criteria represents only 18% of masitinib study patients). The present manuscript reviews >50 CTs conducted in the last 20 years since riluzole was first approved. A special emphasis is put on the analysis of existing evidence in support of the clinical efficacy of edaravone and masitinib and the possible implications of an eventual marketing authorisation in the treatment of ALS.

## Introduction

Amyotrophic lateral sclerosis (ALS) is the most common degenerative disease that affects the motor neuron system. The disease was originally described by Charcot and Joffroy (1869). Despite the nearly century and a half since the condition was first characterized, the extensive accumulated scientific knowledge has not been sufficient to develop truly effective therapeutic strategies to date. ALS primarily affects motor neurons in the brain, brainstem and the spinal cord, causing progressive degeneration and atrophy of voluntary skeletal muscles ultimately resulting in paralysis (Zufiría et al., 2016). The etiology of the disease has proven to be highly complex, undoubtedly contributing to the lack of adequate treatment options. Only about 5–10% of ALS cases are familial (fALS), with a Mendelian pattern of inheritance and at least 13 genes and loci of major effect known to contribute to familial pathology. Of these, toxic gain-of-function mutations in the *SOD1* (superoxide dismutase 1) gene have been most extensively investigated. SOD1 mutations account for approximately 20% of cases of fALS and around 5% of idiopathic/sporadic forms of the disease (sALS) (Kiernan et al., 2011). The exact mechanisms whereby *SOD1* contributes to disease progression are currently unknown. Nevertheless, the generation in 1993 of the first transgenic SOD1^G93A^ mouse model which closely mimics human fALS pathology was paradigmatic as it has allowed the scientific community to investigate ALS disease mechanisms in preclinical species for the first time (Gurney et al., 1994). To this day, rodent SOD1 mutants remain as the most widely employed ALS models to study both the cellular and molecular disease mechanisms and to test the potential efficacy of novel therapeutic compounds (Islam et al., 2014).

Riluzole is the only currently approved treatment option for ALS patients in the US and in Europe, and is only mildly efficacious. The drug received marketing authorisation in 1995 in the USA and in 1996 in Europe. Even though the first large-scale clinical trial (CT) demonstrating clinical efficacy of riluzole to improve survival in ALS patients was conducted in 1990, 3 years prior to the generation of the first *SOD1* mouse model (Bensimon et al., 1994), riluzole has subsequently been shown to have similar efficacy in this transgenic model as seen in human ALS trials (Gurney, 1997), thus validating the preclinical model. It is notable that just like in human patients, riluzole failed to have beneficial effect on disease onset and had only modestly improved survival in these mice. The riluzole data were included in support of the new drug application filed by Rhone-Poulenc-Rorer with the United States Food and Drug Administration (Gurney, 1997). In the years that followed, over 60 molecules have been investigated as a possible treatment for ALS (Mitsumoto et al., 2014; DeLoach et al., 2015). However, the vast majority of pharmaceuticals that reached the CTs stage since riluzole’s approval have failed to demonstrate clinical efficacy.

The aim of the present review is to catalog the results of large-scale CTs that were conducted in the 20 years since riluzole was first approved. An attempt is made to provide a methodical overview of the chosen primary and secondary endpoints with compounds grouped according to their principal mechanism of action. An in-depth analysis is presented for edaravone and masitinib – novel therapeutics that recently demonstrated clinical efficacy in large-scale human CTs in ALS patients.

## Materials and Methods

In May–July 2016, a systematic search for randomized, placebo-controlled clinical trials (RCTs) in ALS patients was performed across the MEDLINE and Clinicaltrials.gov databases (published in English language). In addition, ARISLA list of global ALS trials (ARISLA (Fondazione Italiana de Ricerca Per La Sclerosi Laterale), 2014) was used as a supportive information source.

### Inclusion Criteria

Advanced phase (Phases II, II–III, and III) randomized controlled trials (RCTs) that have recruited ≥100 ALS patients and with the results published between 1995 and July 2016 were included. For the compounds identified in this manner, the search was further expanded to include all the early stage trials that served as a justification for large-scale studies, regardless of the number of patients recruited.

One compound, that did not meet pre-specified inclusion criteria with 63 patients recruited in a Phases 2–3 RCT (memantine; EudraCt # 2007-002117-39) was added to the present analysis as this molecule’s mechanism of action (anti-glutamatergic) is similar to the currently approved riluzole.

## Results

Clinical trials with 24 compounds, in addition to riluzole, are presented in this analysis. Of the 23 molecules in advanced-stage CTs, a total of 51 studies, with a cumulative recruitment of 13,427 ALS patients, are described (**Table 1**).

**Table 1:** List of drugs and compounds tested in human ALS clinical trials.

Compound	Route	Primary endpoint/s	Phase/s	Outcome	Funding^δ^	Reference
***Anti-glutamatergic***						
Ceftriaxone	i.v.	ALSFRS-R; DTP	1–2; 3	Failure	Academic	Berry et al., 2013; Cudkowicz M. et al., 2013
Memantine	Oral	ALSFRS	2–3	Failure	Academic	de Carvalho et al., 2010
Riluzole	Oral	Survival	3	Mixed^∗^	Mixed	Bensimon et al., 1994, 2002; Lacomblez et al., 1996
Talampanel	Oral	ALSFRS-R	2	Failure	Industry	Pascuzzi et al., 2010; Teva, 2010
***Anti-inflammatory***						
Celecoxib	Oral	MVIC	2–3	Failure	Industry	Cudkowicz et al., 2006
Erythropoietin	i.v.	DTP	2; 3	Failure	Academic	Lauria et al., 2009, 2015
Glatiramer acetate	s.c.	ALSFRS-R	2; 2–3	Failure	Industry	Gordon et al., 2006; Meininger et al., 2009
Minocycline	Oral	ALSFRS-R	1–2; 3	Failure	Academic	Gordon et al., 2004, 2007; Pontieri et al., 2005
NP001	i.v. infusion	ALSFRS-R	1; 2	Failure	Industry	Miller et al., 2014, 2015
Pioglitazone	Oral	Survival	2	Failure	Industry	Dupuis et al., 2012
Valproic acid	Oral	DTP	3	Failure	Academic	Piepers et al., 2009
***Anti-oxidative***						
Coenzyme Q10	Oral	ALSFRS-R	2	Failure	Academic	Ferrante et al., 2005; Kaufmann et al., 2009
Creatine	Oral	DTP; MVIC	2; 2–3; 3	Failure	Academic	Rosenfeld, 2001; Groeneveld et al., 2003; Shefner et al., 2004; Rosenfeld et al., 2008; Pastula et al., 2012
Edaravone	i.v. infusion	ALSFRS-R	2; 3	Mixed^∗∗^	Industry	Yoshino and Kimura, 2006; Abe et al., 2014; Palumbo et al., 2016; Sakata et al., 2016; Tanaka et al., 2016a,b
***Neuroprotective***						
Dexpramipexole	Oral	CAFS	2; 3	Failure	Industry	Cudkowicz et al., 2011; Cudkowicz M. E. et al., 2013; Bozik et al., 2014
Olesoxime	Oral	Survival	2–3	Failure	Mixed	Lenglet et al., 2014
TCH346	Oral	ALSFRS-R	2–3	Failure	Industry	Miller et al., 2007a
Xaliproden	Oral	MMT; DTP	2; 3	Failure	Industry	Lacomblez et al., 2004; Meininger et al., 2004
***Neurotrophic factors***						
BDNF	i.v.	Survival; %FVC	1–2; 3	Failure	Mixed	Bradley, 1995; Kasarskis et al., 1999
CNTF	s.c.	MVIC	1; 1–2; 2–3; 3	Failure	Mixed	Miller et al., 1993, 1996; ALS CNTF Treatment Study Group, 1995, 1996
IGF-1	s.c.	AALS; MMT	3	Failure	Mixed	Lai et al., 1997; Borasio et al., 1998; Sorenson et al., 2008
***CSF1R inhibition***						
Masitinib	Oral	ALSFRS-R	2–3	Positive^$^	Industry	
***Other***						
Lithium	Oral	ALSFRS-R; Survival	2; 2–3; 3	Failure^£^	Mixed	Fornai et al., 2008; Aggarwal et al., 2010; Verstraete et al., 2012; Morrison et al., 2013
Tirasemtiv	Oral	ALSFRS-R	2	Failure	Industry	Shefner et al., 2012, 2013a,b, 2016

*^∗^: Two out of three late-stage trials reported negative results; ^∗∗^: two out of three Phase 3 trials reported negative results; $: statistical significance reached on multiple efficacy endpoints based on the results of interim analysis with 192 patients (50% of enrolled patients); £: only one pilot early phase trial reported statistical significance on efficacy endpoints, all subsequent large-scale follow-up studies failed to reproduce these positive results.*

*δ: Reported funding source, as described by the authors of relevant publications.*

*AALS, Appel ALS Rating Scale; ALSFRS-(R), The ALS Functional Rating Scale (Revised); CAFS, combined assessment of function and survival; DTP, time to death, tracheostomy or persistent assisted ventilation; %FVC, forced vital capacity; MMT, manual muscle test; MVIC, maximum voluntary isometric contraction; CSF1R, colony stimulating factor 1 receptor; i.m., intramuscular i.v., intravenous; s.c., subcutaneous.*

A wide range of compounds, representing at least 10 different broadly defined mechanisms of action met the pre-specified inclusion criteria. This both reflects on the disease complexity and highlights the intensive research efforts put forth by the academic scientists and the pharmaceutical industry.

Revised ALS Functional Rating Scale (ALSFRS-R) was chosen as the primary endpoint in CTs with 11 (48%) of the 23 drugs in advanced clinical development. 15 (65%) of compounds are administered *per os*, 3 (13%) subcutaneously (*s.c*.), 3 (13%) intravenously (*i.v.*), and a further 2 (9%) drugs via continuous *i.v.* infusion.

Only two RCTs, which included pre-specified efficacy measures as the primary endpoint and recruited ≥100 patients, were successful on the primary endpoint. Based on the results of pre-specified interim analysis, oral masitinib has shown therapeutic benefit on the primary endpoint (ALSFRS-R), as well as secondary endpoints of forced vital capacity (FVC), combined assessment of function and survival (CAFS), and ALSAQ-40 (40-items ALS quality of life assessment questionnaire) (AB Science, press-release www.ab-science.com/file_bdd/content/1475163914_ABSCIENCESLAEMAFilingvEngVF.pdf) – in a broad ALS patient population in advanced stage Phases 2–3 RCT. The study has recently completed recruitment (392 patients), with the results of the final analysis expected to be available in 2017 (trial ID# NCT02588677). Edaravone is a second drug that had shown clinical efficacy on the primary endpoint (ALSFRS-R) and ALSAQ-40 secondary endpoint in one of the three Phase 3 RCTs (trial ID# NCT01492686), recruiting 137 patients (Tanaka et al., 2016a). The other two RCTs with edaravone in ALS, which recruited patients with a wider inclusion criteria (Abe et al., 2014), and patients with more advanced disease (Tanaka et al., 2016b) have failed to reach statistical significance on the primary endpoint. Edaravone treatment regimen requires 2-weeks on, 2 weeks-off daily continuous i.v. infusions (30 min) of the drug substance, a factor which needs to be taken into consideration when assessing quality of life. In terms of patient inclusion, CT with masitinib recruited a wider, more representative, less restrictive patient population in comparison to the only successful edaravone CT (edaravone eligibility criteria represents only 18% of masitinib study patients).

Only one early stage trial (lithium), which included a comparator arm and recruited <100 patients was successful on the primary efficacy endpoint (Fornai et al., 2008). The limitation of that study was that it recruited only 16 patients in the treatment arm and 28 in the active control arm, randomization methods were not clearly described, and the study utilized an open-label design. All subsequent confirmatory RCTs with lithium have failed to show evidence of clinical benefit (Aggarwal et al., 2010; Verstraete et al., 2012; Morrison et al., 2013).

Of the 31 studies that included pre-specified efficacy measures as the secondary endpoint(s) and recruited ≥100 patients, 26 studies did not reach statistical significance in any of the secondary endpoints in the pre-defined analyses (including studies that did not report *P*-values).

One study [Xaliproden (Lacomblez et al., 2004)] has reached statistical significance in only one of the eight secondary endpoints, and at only one of the two doses assessed. The study failed on the primary endpoint. One study [Tirasemtiv (Shefner et al., 2016)] has reached statistical significance in two of the five secondary endpoints. The study failed on the primary endpoint. One study [IGF-1 (Lai et al., 1997)] has reached statistical significance for the high-dose group in three out of three secondary endpoints. The study failed on the primary endpoint. Authors acknowledge that the treatment effect was very modest and that the results of the study were inconclusive.

The other two compounds that reached statistical significance on secondary endpoints are masitinib and edaravone. Both of these drugs have also reported statistically significant treatment effect on primary endpoints.

Detailed descriptions of the study designs and the treatment outcomes for the CTs presented in **Table 1** are available as Supplemental Materials. As a significant number of original publications do not provide comprehensive details on the way the patient discontinuations are handled in study protocols, such data is not presented as a part of the present Review.

## Discussion

The analysis of CTs presented in this systematic review is a testament to the extraordinary complexity of the ALS disease mechanisms. Even though academic science has made significant progress in attempting to elucidate molecular, genetic, epigenetic and environmental aspects of motor neuron degeneration, the slow translational progress with only one approved drug on the US and European markets suggests that existing preclinical models are not fully representative of human disease process. The absence of the fully characterized and validated rodent ALS models is increasingly recognized as a major hurdle in clinical development. In a recent study by ALS Therapy Development Institute (ALS TDI), researchers performed rigorous animal tests on multiple compounds which failed in CTs. Surprisingly, the improvement in survival which was previously published for all those drug candidates (including riluzole) could not be replicated in an ALS TDI study (Perrin, 2014). Authors emphasize the need for community effort in the area of preclinical model development both by the public and private agencies. In the meantime, currently available preclinical models should be considered useful only to a certain extent, with the ultimate test of the potential efficacy of any novel pharmaceutical having to come from large-scale human CTs.

Up-to-date analysis of the state of the current understanding of the molecular and other aspects of preclinical ALS research had been presented in a number of recent review papers (Belzil et al., 2016; Bozzoni, 2016; Clerc et al., 2016; Riva et al., 2016; Therrien et al., 2016; Zufiría et al., 2016). While genetic mutations in SOD1 gene undeniably contribute to the course of familial ALS (fALS), it is only recently that we are beginning to uncover additional factors that play a role in the etiology of sporadic ALS. Taking into account that only about 10% of all patients present with a fALS, with the remaining 90% of sufferers presenting with sALS (Riva et al., 2016), it is of utmost importance that novel therapeutics show clinical efficacy in sALS patient population. Furthermore, recent epidemiological evidence suggests an existing variation of ALS incidence between subcontinents that might be related to population ancestries. While homogenous incidence rates have been reported in populations from Europe, North America and New Zealand, with pooled ALS standardized incidence of 1.81 (1.66–1.97) per 100,000 person-years of follow-up (PYFU), Asian populations appear to be distinct. Differences were identified in ALS standardized incidence between North Europe [1.89 (1.46–2.32)/100,000 PYFU] and East Asia [0.83 (0.42–1.24)/100,000 PYFU, China and Japan, *P* = 0.001] and South Asia [0.73 (0.58–0.89)/100,000 PYFU, Iran, *P* = 0.02] (Marin et al., 2016). Such geographical variation is an important factor that needs to be taken into account when conducting global CTs and, for example, a hypothetical compound showing clinical benefit in Asian population may not necessarily be efficacious in European and North American populations.

The following sections provide a brief overview of human CTs with the molecules presented in **Table 1** and Supplementary Materials, grouped according to the putative mechanism of action. A more in-depth discussion of the CT outcomes for the two promising therapeutics, which claim potential clinical efficacy in human CTs (edaravone and masitinib), is presented in the latter part of this manuscript.

### Anti-glutamatergic

It is thought that glutamate excitotoxicity plays a role in the ALS pathogenesis (Rothstein, 1995). Glutamate is the principal excitatory neurotransmitter in the central nervous system. It is hypothesized that aberrant glutamatergic system overactivation may lead to damage and an eventual death of the motor neurons in ALS (Rothstein et al., 2005). Riluzole is the only currently approved ALS treatment in the US and in Europe, which moderately increases ALS patient survival (by 2–3 months). The first-in-human CT with riluzole was launched on the basis of the hypothesis that riluzole may modulate glutamatergic transmission, which was supported by pre-clinical data (Bensimon et al., 1994). Extensive research into the mechanisms of action of riluzole have since then consistently suggested that direct effects of riluzole on glutamate receptors are limited, and usually require high concentrations, which are not achieved in human patients. It is becoming increasingly clear that the molecular effects of riluzole are manifold and highly complex, including the modulation of persistent Na^+^ channel current, potentiation of calcium-dependent K^+^ current, inhibition of neurotransmitter release, inhibition of fast Na^+^ current, inhibition of voltage-gated Ca^2+^ current and other effects on neurotransmission (reviewed by Bellingham, 2011). It is this complexity of neural effects of riluzole, which may explain the failure in the CTs of other molecules which have targeted glutamatergic transmission as their primary mechanism of action (ceftriaxone, memantine, and talampanel). Despite the absence of the clear consensus on the exact mechanism of action of riluzole in ALS, the drug narrowly received marketing authorisation by the FDA based on the results of three late-stage CTs, recruiting over 1000 patients (Bensimon et al., 1994, 2002; Lacomblez et al., 1996; Miller et al., 2007b).

Ceftriaxone is the only intravenous beta-lactam antibiotic which was investigated in a large-scale non-stop Phases 1–3 RCT recruiting 513 ALS patients (Berry et al., 2013; Cudkowicz M. et al., 2013). The study was launched on the basis of existing evidence that beta-lactam antibiotics may have neuroprotective (anti-excitotoxic) effects in a number of *in vitro* and in *in vivo* preclinical models of ALS (Rothstein et al., 2005). Ceftriaxone administration results in the upregulation of the glutamate transporter GLT1, the primary molecule responsible for synaptic glutamate inactivation. The drug delayed neuronal and muscle strength loss, as well as increased survival in mouse ALS models (Rothstein et al., 2005). The study failed to demonstrate significant improvement in survival and functional outcomes in ALS patients (Berry et al., 2013; Cudkowicz M. et al., 2013). Similar rationale was used to launch a moderate-size CT with memantine, a novel moderate-affinity voltage-dependent non-competitive antagonist at glutamatergic NMDA receptors, which preserves the physiological function of the receptor. Memantine is currently approved for the treatment of Alzheimer disease. Just like ceftriaxone, memantine prolonged survival in a SOD1^G93A^ mouse model of ALS (Wang and Zhang, 2005). A RCT with 63 patients failed to demonstrate any treatment effect in ALS patients (de Carvalho et al., 2010). Talampanel is another compound targeting glutamatergic transmission that failed in advanced-stage CTs in ALS patients. Only the results of the failed Phase 2 study with 58 patients were published in a peer-reviewed journal (Pascuzzi et al., 2010). The data for the failed Phase 3 study, recruiting 559 patients (trial ID# NCT00696332) was not publicly disclosed (Supplementary Materials).

### Anti-inflammatory

Neuroinflammation is a prominent pathological finding in ALS patients and is characterized by microglial activation, astrogliosis, and infiltration of monocytes and T-cells. Neuroinflammatory responses can be beneficial or harmful to motor neuron survival. The release of signals from motor neurons represents the earliest stages of ALS with microglia responding as an M2 phenotype, releasing neuroprotective factors to repair motor neurons and protect against further injury. As disease progresses, however, the balance of neuroinflammatory mediators changes leading to the exacerbation of the disease phenotype (Zhao et al., 2013). Therapeutic regimens with an anti-inflammatory mechanism of action may thus be of potential benefit to the ALS sufferers. Seven compounds with primarily anti-inflammatory MoA are presented in this manuscript. Unfortunately, all CTs with these therapeutics have failed to show improvement on function in ALS patients. All seven therapeutics, which include celexocib (Cudkowicz et al., 2006), erythropoietin (Lauria et al., 2009, 2015), glatiramer acetate (Gordon et al., 2006; Meininger et al., 2009), minocycline (Gordon et al., 2004, 2007; Pontieri et al., 2005), NP001 (Miller et al., 2014, 2015), pioglitazone (Dupuis et al., 2012), and valproic acid (Piepers et al., 2009) have demonstrated preclinical efficacy in the SOD1 rodent models of ALS. The failure of advanced-stage CTs has resulted in the termination of the clinical development in ALS for all of the above mentioned pharmaceuticals.

### Anti-oxidative

The excessive production of reactive oxygen species and the resultant oxidative stress and insufficient activity of antioxidant defense mechanisms have been implicated in the pathogenesis of many neurodegenerative diseases, including ALS (Li et al., 2013). The involvement of the oxidative signaling pathways in ALS pathology was considered as possible contributor to disease course early on. As mentioned earlier, SOD1 transgenic models are the most widely studied preclinical models of the disease. Mutated SOD1 can form cytotoxic protein aggregates, possibly leading to the loss of enzymatic function, or mutated SOD1 may acquire cytotoxic properties (toxic gain-of-function) (Saccon et al., 2013). As SOD1 plays a key role in reactive oxygen clearance, defective enzymatic activity may potentially lead to increased levels of oxidative stress (Niedzielska et al., 2015). The presence of oxidative stress biomarkers was also previously confirmed in the cerebrospinal fluid of living ALS patients (Tohgi et al., 1999). Of the three compounds possessing anti-oxidative properties described in this review, two compounds have failed to demonstrate clinical efficacy in large CTs: creatine (Rosenfeld, 2001; Groeneveld et al., 2003; Shefner et al., 2004; Rosenfeld et al., 2008; Pastula et al., 2012) and coenzyme Q10 (Ferrante et al., 2005; Kaufmann et al., 2009). Edaravone is the only anti-oxidative treatment which have demonstrated clinical efficacy on the primary endpoint (ALSFRS-R) in one of the three Phase 3 CTs, which recruited 137 patients. The results of this trial were presented as a conference abstract, and the complete data had not been published as a full paper at the time of the submission of this manuscript (Tanaka et al., 2016a). Nevertheless, edaravone has received marketing authorisation exclusively in Japan in 2015. A detailed overview of the clinical development of edaravone is provided below in a separate section.

Creatine is a naturally occurring nitrogenous organic acid found predominantly in the skeletal muscle where it contributes to the production of adenosine triphosphate during times of anaerobic metabolism (Bessman, 1987). It appears to possess antioxidant and neuroprotective properties and may improve mitochondrial function (Adhihetty and Beal, 2008). The publication of a report in 1999 that creatine administration in SOD1^G93A^ mutant mice produced a dose-dependent improvement in motor performance and extended survival (Klivenyi et al., 1999) has resulted in launch of a number of large-scale human trials in ALS patients, both in the US and in Europe (Groeneveld et al., 2003; Shefner et al., 2004; Rosenfeld et al., 2008; Pastula et al., 2012). No clinical benefit was demonstrated in any of human trials. Coenzyme Q10 is an essential cofactor of the electron transport chain as well as an important antioxidant, which is particularly effective within mitochondria. Coenzyme Q10 administration also significantly extended survival in an ALS mouse model (Beal, 2002), the results which did not translate into success in human trials, which all failed (Ferrante et al., 2005; Kaufmann et al., 2009).

### Neuroprotective

Amyotrophic lateral sclerosis is a neurodegenerative disease which results in the eventual destruction of the motor neuron. Compounds which are potentially neuroprotective are thus hypothesized to have an appreciable clinical benefit in ALS patients. It is perhaps not surprising then, that therapeutics that fall into this category have recruited very large patient populations in their respective Phase 3 trials. CTs with olesoxime (Lenglet et al., 2014) and TCH346 (Miller et al., 2007a) have recruited >500 patients each, with dexpramipexole (Cudkowicz et al., 2011; Cudkowicz M. E. et al., 2013) and xaliproden (Lacomblez et al., 2004; Meininger et al., 2004) recruiting >1000 and >2000 patients respectively (Supplementary Materials).

Dexpramipexole belongs to a class of compounds termed benzothiazoles, structurally uncomplicated molecules with a broad range of biological activity. In the central nervous system, dexpramipexole has demonstrated mitochondrial function-dependent neuroprotective activity in preclinical models, including modest improvement in survival in the SOD1^G93A^ mouse model of ALS (Gribkoff and Bozik, 2008), which served as a rationale for initiating clinical development. The compound failed to demonstrate clinical benefit in ALS (Cudkowicz et al., 2011; Cudkowicz M. E. et al., 2013).

Olesoxime is a cholesterol-like small molecule with the neuroprotective mechanism thought to be dependent on mitochondria. In line with the reoccurring pattern plaguing ALS clinical development, the molecule improved motor performance, delayed the onset of clinical disease, and extended survival in SOD1^G93A^ transgenic mice (Bordet et al., 2007), but failed in the CT (Lenglet et al., 2014).

TCH346 is a small molecule which can be neuroprotective as a result of the inhibition of both the apoptotic increases of the glycolytic enzyme, glyceraldehyde 3-phosphate dehydrogenase (GAPDH), and the nuclear accumulation of GAPDH (Carlile et al., 2000). It is one the few compounds (together with xaliproden), human clinical development of which was not initiated based on positive data in SOD1 mutant models. Rather, TCH346 treatment has demonstrated decreased loss of motoneurons and motoneuron fibers in a mouse mutant model of progressive motor neuronopathy (PMN), a hereditary autosomal recessive wasting disease which shares some symptoms of ALS (Sagot et al., 2000). Subsequent large-scale CT in human ALS patients had failed to demonstrate clinical efficacy on any of the functional endpoints (Miller et al., 2007a). Xaliproden is a non-peptide compound of low molecular weight, which was observed to promote the secretion or stimulate receptors of several endogenous growth factors active on motor neurons, thus conferring neuroprotection (Lacomblez et al., 2004). Like TCH346, xaliproden delayed disease progression and improved survival in a PMN mouse model (Duong et al., 1998). The two Phase 3 CTs presented in current review are also the largest, with the cumulative recruitment of >2000 patients. Both studies featured identical design and were conducted in ALS patients with (Study 2) or without (Study 1) background riluzole therapy (Meininger et al., 2004). Both studies failed on primary endpoints resulting in termination of clinical development in ALS.

### Neurotrophic Factors

Many neurotrophic factors are known to promote neuronal survival and regeneration. As a result, a large number of these factors have been investigated in preclinical models of ALS. These include BDNF, CNTF, GDNF, IGF-1, VEGF, FGF; HGF, BMP-7, and G-CSF (for an excellent review of neurotrophic factors both in preclinical development and in CTs in ALS refer to Henriques et al. (2010). Three factors were investigated in large-scale human CTs. All failed to show improvement in human ALS patients. CTs with brain-derived neurotrophic factor (BDNF) (Bradley, 1995; Kasarskis et al., 1999), ciliary neurotrophic factor (CNTF) (Miller et al., 1993, 1996; ALS CNTF Treatment Study Group, 1995, 1996) and insulin-like growth factor I (IGF-1) (Lai et al., 1997; Borasio et al., 1998; Sorenson et al., 2008) are presented in **Table 1** and Supplementary Materials.

### Other

#### Lithium

Lithium is a special case in the history of the ALS CTs. It is a compound used as a mood stabilizer and may have potential neuroprotective properties. In an unusual report, which published both the results of lithium treatment in SOD1^G93A^ mice and the results of a pilot human trial in the same publication, authors found a remarkable improvement of survival in human patients (Fornai et al., 2008). In a human trial which randomized 16 ALS patients into lithium + riluzole treatment arm versus 28 patients in the control arm (riluzole alone), no deaths were reported in the treatment arm versus eight deaths (29%) in the riluzole arm after a 15 months treatment period. Furthermore, remarkable improvement was also reported for a number of secondary functional endpoints. Understandably, the results of this study generated considerable interest in the potential of lithium as a treatment of ALS. No less than three CTs were quickly launched by independent research groups in order to confirm the results in double-blind randomized placebo-controlled studies (the original study utilized an open-label design). Unfortunately, all subsequent studies (Aggarwal et al., 2010; Verstraete et al., 2012; Morrison et al., 2013) have failed to replicate the positive results of the pilot trial. Lithium fiasco is a cautionary tale, highlighting the importance of the use of appropriately chosen patient populations, especially in a disease as difficult to treat as ALS.

#### Tirasemtiv

Tirasemtiv is an orally bioavailable molecule that sensitizes the troponin complex in fast-twitch skeletal muscle fibers to calcium. A putative mechanism of action is an improvement in the contraction of muscle in response to diminished neural input thus potentially decreasing muscle fatigue (Shefner et al., 2012). As muscle wasting is a symptom of ALS, a number of human trials were launched in ALS patients (Shefner et al., 2012, 2013a,b, 2016). The only large-scale Phase 2b study which used a functional measure (ALSFRS-R) as the primary endpoint and recruited 605 patients has failed on the primary endpoint (Shefner et al., 2016). Because the study has shown statistical significance on two out of five secondary endpoints (slow vital capacity and muscle strength), the authors leave open the possibility to potentially launch an additional Phase 3 study in the future.

### Edaravone

Edaravone is an intravenous free radical scavenger which eliminates lipid peroxide and hydroxyl radicals and can thus be classified as an anti-oxidative agent (Watanabe et al., 1994). As discussed earlier, oxidative stress is thought to contribute to ALS pathology. Beneficial effects of edaravone have been demonstrated in a preclinical model in wobbler mice with ALS-like symptoms prior to the initiation of clinical development (Yoshino and Kimura, 2006). First pilot CT of edaravone in ALS employed an uncontrolled open-label design, did not use a comparator arm and recruited a total of 19 patients randomized into two dosage groups, with five patients receiving 30 mg daily i.v. infusions, and the remaining 14 patients receiving 60 mg daily i.v. infusions on a 2-weeks-on, 2-weeks-off schedule. Change in ALSFRS-R was designated as the primary endpoint. Study utilized an atypical design with the treatment effect calculated on the basis of the functional decline on ALSFRS-R in the same patients in the 6 months prior to treatment compared to changes observed following 6 months of treatment. Nevertheless, statistically significant treatment effect was observed on the primary endpoint in the high-dose group.

The first confirmatory large-scale Phase 3 trial was launched on the basis of the results of the pilot trial (Abe et al., 2014). The study was an RCT which recruited a total of 206 patients (placebo: 104; edaravone: 102). Patient inclusion criteria included FVC of at least 70% and the time since disease onset of <3 years, as well as allowed inclusion of patients with probable laboratory-supported El Escorial criteria. ALSFRS-R was designated as the primary endpoint. The study has failed to demonstrate statistically significant treatment effect in any of the functional endpoints. However, subgroup analysis revealed not-statistically significant differences when patients were separated into three subgroups according to the severity of the disease progression. A trend was noted that edaravone appeared to show possible treatment effect in patients with most severe and moderate disease progression while no treatment effect was observed in patients with least severe disease. Based on these results, two additional confirmatory Phase 3 studies were launched. At the time of the submission of this manuscript, the results of the 2nd and 3rd confirmatory studies were presented as conference abstracts only and have not yet been published in peer-review sources.

The second confirmatory Phase 3 study (trial ID#: NCT00415519) recruited 25 patients with severe ALS, with the inclusion criteria of FVC of at least 60% and the time since disease onset of <3 years. The study failed to demonstrate any treatment effect between placebo and edaravone arms (Tanaka et al., 2016b).

The third confirmatory Phase 3 (Tanaka et al., 2016a) study (trial ID#: NCT01492686) narrowed the inclusion criteria even further (when compared to the first confirmatory study) and excluded both the patients with more severe disease and the patient population with the least severe disease (patients with probable laboratory-supported ALS were no longer part of inclusion criteria). Inclusion criteria included FVC of at least 80% and the time since disease onset of <2 years. This third confirmatory Phase 3 trial recruiting 137 ALS patients was successful on the primary endpoint of ALSFRS-R, as well as on ALSAQ-40 quality-of-life questionnaire. Mean change (±SE) in ALSFRS-R score was -7.50 ± 0.66 (placebo) and -5.01 ± 0.64 (edaravone): a between group difference of 2.49 ± 0.76 (*P* = 0.001). Between group difference for ALSAQ40 was -8.79 ± 4.03 (*P* = 0.031). On the basis of the results of the only successful confirmatory Phase 3 trial, edaravone received marketing authorisation exclusively in Japan in 2015.

It is important to note that all CTs with edaravone were conducted in Japanese population. As mentioned earlier, epidemiological studies point to the existence of significant difference in ALS incidence in populations of Asian versus European/North American descents (Marin et al., 2016). If edaravone is to become eventually available to the ALS sufferers in Western countries, it will be beneficial to see a large-scale CT with edaravone recruiting ALS patients of Western ancestry.

Patient burden can be considered as an additional factor which should be taken into consideration. Edaravone treatment requires continuous 30 min-long daily intravenous infusions on a 2-weeks-on, 2-weeks-off schedule for an indefinite time period. For a disease as severe as ALS this may be deemed acceptable, however, in some markets such a route of administration may require a hospital setting.

### Masitinib

Masitinib is a highly selective tyrosine kinase inhibitor which was previously shown to prevent CNS neuroinflammation in a number of neurodegenerative disease models including in ALS SOD1^G93A^ rats (Piette et al., 2011; Vermersch et al., 2012; Kocic et al., 2015; Trias et al., 2016). In a SOD1^G93A^ rodent model, masitinib significantly prolonged survival when delivered after paralysis onset, an unprecedented effect in preclinical models of ALS. Masitinib was also shown to be capable of controlling microgliosis, neuroinflammation, and the emergence/expansion of aberrant glial cells in preclinical ALS model. Masitinib’s mechanism of action can thus be considered unique among the compounds described in this review (Trias et al., 2016).

Masitinib is currently undergoing Phase 3 development in ALS patients as an add-on to riluzole therapy (trial ID# NCT02588677), recruiting 382 patients into three treatment arms (placebo versus 3.0 and 4.5 mg/kg/day oral masitinib, with a 1:1:1 randomization). Patient recruitment has been completed at the time this manuscript was submitted. A planned interim analysis was carried out when 50% (191) patients had completed 48 weeks of treatment, with ALSFRS-R as the primary endpoint. Results demonstrated success on the primary ALSFRS-R endpoint (with a significance level of *P* < 0.01) in the intent-to-treat population, as well as significance in numerous sensitivity analyses of the primary endpoint (AB Science, unpublished). Of note, the study employed relatively broad patient inclusion criteria, including patients with FVC of >60% and time since disease onset of less <3 years. When these and other inclusion criteria are taken into consideration, masitinib appears to have included a far wider subgroup of ALS patients when compared with the only successful edaravone confirmatory CT (the overall edaravone subpopulation represents only 18% of patients that would have been eligible for the masitinib trial).

The study will be completed when 100% of the enrolled patients have completed the 48-week treatment protocol period. Final data readout is expected before mid-2017. Considering the near total failure of ALS CTs over the last 20 years, these results are encouraging and the prospects of an eventual marketing authorization are currently being explored (AB Science Press Release; www.ab-science.com/file_bdd/content/1475163914_ABSCIENCESLAEMAFilingvEngVF.pdf).

It will be of interest to evaluate possible clinical potentials of edaravone versus masitinib. Full publication of results from clinical studies with both compounds in ALS will be of great interest so that a more rigorous comparison of efficacy and safety between these promising candidate treatments can be made. However, given the information currently available and assuming equivalence in all other aspects (e.g., efficacy, safety, and cost), masitinib would appear to provide clinical advantages over edaravone in terms of level of patient burden, which probably translates to patient compliance. Furthermore, the only successful edaravone CT have used strict inclusion criteria, with the clinical benefit demonstrated in a population representative of only 18% of ALS patients included in the masitinib trial. It will also be of interest to see a clinical study of edaravone in ALS patients of Western ancestries. Because this two drugs have different mehanisms of action, it will be of interest to test whether or not they may exhibit synergistic effects in animal models and ultimately in CT in humans.

### Non-pharmacologic and Symptomatic Disease Management

In view of the current absence of adequate pharmacotherapy for ALS patients and the fact that regulatory approval can take years even for promising drug candidates, alternative treatment options should not be overlooked. In recent years, stem cell therapy has emerged as potential non-pharmacological new treatment for patients with ALS. It is hypothesized that stem cells may support dying motor neurons though reducing inflammation, releasing growth factors, and other potential mechanisms (Thomsen et al., 2014). Several pre-clinical and CTs have used human bone marrow stromal-derived mesenchymal stem cells (MSCs) that are differentiated into specialized neuron-supporting cells to stably secrete neurotrophic factors. They have been shown to exhibit MSC markers, display spindle-like cell morphology, form single-cell-derived colonies, readily differentiate into adipocytes and osteoblasts, and have the capacity to express a number of neural genes (Blondheim et al., 2006). To date, there are no peer-reviewed, proof-of-concept studies using these cells in an ALS animal model and therefore it is difficult to make assessments on the pre-clinical rationale for their use in the treatment of ALS (Thomsen et al., 2014).

Multidisciplinary symptomatic care can lead to significant improvement in the quality of life of individual patients. However, supportive and symptomatic care does not usually fall within the scope of primary endpoint analyses in well-controlled RCTs, causing physicians to base their treatment opinions on personal experience or literature reports which are frequently based on anecdotal data (Jackson et al., 2015). Academic and medical communities are increasingly recognizing the need for evidence-based guidelines regarding the symptomatic care in ALS patients including the management of such issues as respiratory failure, nutrition, pain, fatigue, and cognitive impairment. A number of palliative treatment options designed to deal with the multiplicity of ALS symptoms have recently been reviewed (Hobson and McDermott, 2016). Nevertheless, additional well-controlled studies with the primary aim of improving the quality-of-life of ALS patients will be welcome.

## Conclusion

Nearly all of the CTs since riluzole was first approved have failed to demonstrate clinical efficacy in the treatment of ALS. But now, for the first time in the past 20 years, there is real encouragement that we may soon have a new and more effective treatment option available, pending approval from regulatory agencies. Two potentially efficacious treatments are claiming to demonstrate clinical benefit on functional endpoints in human CTs. Masitinib and edaravone have passed the first significant milestones on the road to potential approval in the US and European markets. Even though edaravone already received marketing authorisation in Japan based on mixed results from multiple CTs in Japanese patients, the only successful CT with edaravone recruited a restrictive ALS patient population. When compared to masitinib, the edaravone trial targeted only around 18% of ALS patients who would have been eligible to the masitinib trial. In case of masitinib, the majority of recruited patients are of Western ancestry, but claimed clinical efficacy is so far based on the results of the successful unpublished interim analysis demonstrating encouraging results on multiple study endpoints. However, the study has already completed patient recruitment and full study results are expected in 2017. Authors are cautiously optimistic that one or more of the above mentioned treatments will become available to ALS patients in the US and Europe in the near future.

## Author Contributions

DP wrote the manuscript. AM, CM, and OH performed content revisions, significantly contributed to data analysis and interpretation, and proofread the manuscript. All authors take the responsibility for the accuracy and integrity of the presented work.

## Conflict of Interest Statement

AM, CM, and DP are employees of AB Science. OH has received financial contributions from AB Science.
