# Supplementary material for: ALS Clinical Trials Review: 20 Years of Failure. Are We Any Closer to Registering a New Treatment?
Source: Front Aging Neurosci. 2017 Mar 22;9:68. doi: 10.3389/fnagi.2017.00068 (PMC5360725; doi:10.3389/fnagi.2017.00068)
Supplement: Supplementary file 1 [file Data_Sheet_1.docx]

**SUPPLEMENTARY MATERIALS**

Brief overview of the primary and secondary endpoints and outcomes of ALS clinical trials mentioned in the manuscript

Table of Contents

[r-metHuBDNF 5](#_Toc457213774)

[BDNF Study Group PI-II (Bradley, 1995) 5](#_Toc457213775)

[BDNF Study Group PIII (Kasarskis et al., 1999) 6](#_Toc457213776)

[Ceftriaxone 8](#_Toc457213777)

[NCT00349622 – Stage I-II (Berry et al., 2013) 8](#_Toc457213778)

[NCT00349622 – Stage III (Cudkowicz et al., 2013b) 9](#_Toc457213779)

[Celexocib 10](#_Toc457213780)

[NEALS (Celexocib) (Cudkowicz et al., 2006) 10](#_Toc457213781)

[rhCNTF 11](#_Toc457213782)

[ALS CNTF Study Group (ALS CNTF Treatment Study Group, 1996) 11](#_Toc457213783)

[rhCNTF Study Group (Miller et al., 1996) 13](#_Toc457213784)

[Coenzyme Q10 14](#_Toc457213785)

[Ferrante 2005 (Kaufmann et al., 2009) 14](#_Toc457213786)

[NCT00243932 – Stage 1 (Kaufmann et al., 2009) 15](#_Toc457213787)

[NCT00243932 – Stage 2 (Kaufmann et al., 2009) 16](#_Toc457213788)

[Creatine 17](#_Toc457213789)

[Groeneveld 2003 (Groeneveld et al., 2003) 17](#_Toc457213790)

[NCT00005674 (NEALS) (Pastula et al., 2012; Shefner et al., 2004) 18](#_Toc457213791)

[NCT00069186 (Pastula et al., 2012; Rosenfeld, 2001; Rosenfeld et al., 2008) 19](#_Toc457213792)

[Dexpramipexole 20](#_Toc457213793)

[NCT00647296 – Part 1 (Cudkowicz et al., 2011) 20](#_Toc457213794)

[NCT00647296 – Part 2 (Cudkowicz et al., 2011) 21](#_Toc457213795)

[NCT01281189 (EMPOWER) (Bozik et al., 2014; Cudkowicz et al., 2013a) 22](#_Toc457213796)

[Edaravone 23](#_Toc457213797)

[Yoshino 2006 (Yoshino and Kimura, 2006) 23](#_Toc457213798)

[NCT00330681 (Abe et al., 2014) 24](#_Toc457213799)

[Erythpoietin 26](#_Toc457213800)

[EudraCT # 2005-005873-31 (Lauria et al., 2009) 26](#_Toc457213801)

[EudraCT # 2009-016066-91 (Lauria et al., 2015) 28](#_Toc457213802)

[Glatiramer acetate 29](#_Toc457213803)

[Gordon 2006 (Gordon et al., 2006) 29](#_Toc457213804)

[EudraCT (2006-001688-49) (Meininger et al., 2009) 30](#_Toc457213805)

[r-Hu-IGF-1 31](#_Toc457213806)

[Lai 1997 (Lai et al., 1997) 31](#_Toc457213807)

[Borasio 1998 (Borasio et al., 1998) 33](#_Toc457213808)

[NCT00035815 (Sorenson et al., 2008) 34](#_Toc457213809)

[Lithium 35](#_Toc457213810)

[Fornai 2008 (Fornai et al., 2008) 35](#_Toc457213811)

[NCT00818389 - (NEALS) (Aggarwal et al., 2010) 37](#_Toc457213812)

[NTR1448 - (Netherlands) (Verstraete et al., 2012) 38](#_Toc457213813)

[LiCALS - EudraCT (2008-006891-31) (Morrison et al., 2013) 39](#_Toc457213814)

[Masitinib 40](#_Toc457213815)

[Memantine 41](#_Toc457213816)

[NCT00353665 (de Carvalho et al., 2010) 41](#_Toc457213817)

[Minocycline 43](#_Toc457213818)

[Gordon 2004 (Gordon et al., 2004) 43](#_Toc457213819)

[Pontieri 2005 (Pontieri et al., 2005) 45](#_Toc457213820)

[NCT00047723 (Gordon et al., 2007) 46](#_Toc457213821)

[NP001 47](#_Toc457213822)

[NCT01091142 (Miller et al., 2014) 47](#_Toc457213823)

[NCT01281631 (Miller et al., 2015) 48](#_Toc457213824)

[Olesoxine 49](#_Toc457213825)

[NCT00868166 (Lenglet et al., 2014) 49](#_Toc457213826)

[Pioglitazone 50](#_Toc457213827)

[NCT00690118 (Dupuis et al., 2012) 50](#_Toc457213828)

[Talampanel 51](#_Toc457213829)

[Pascuzzi 2010 (Pascuzzi et al., 2010) 51](#_Toc457213830)

[NCT00696332 (Teva, 2010) 52](#_Toc457213831)

[TCH346 53](#_Toc457213832)

[NCT00072709 (Miller et al., 2007) 53](#_Toc457213833)

[Tirasemtiv 54](#_Toc457213834)

[NCT01089010 (Shefner et al., 2012) 54](#_Toc457213835)

[NCT01378676 (Parts 1 and 2) (Shefner et al., 2013a) 56](#_Toc457213836)

[NCT01378676 (Part 3) (Shefner et al., 2013a) 58](#_Toc457213837)

[Phase II meta-analysis (Shefner et al., 2013b) 59](#_Toc457213838)

[NCT01709149 (Shefner et al., 2016) 61](#_Toc457213839)

[Valproic acid 63](#_Toc457213840)

[NCT00136110 (Piepers et al., 2009) 63](#_Toc457213841)

[Xaliproden 64](#_Toc457213842)

[Lacomblez – PII (Lacomblez et al., 2004) 64](#_Toc457213843)

[EFC2941 (Meininger et al., 2004) 66](#_Toc457213844)

[EFC1923 (Meininger et al., 2004) 68](#_Toc457213845)

[Bibliography 70](#_Toc457213846)

# r-metHuBDNF

## BDNF Study Group PI-II (Bradley, 1995)

**Compound:** r-metHuBDNF

**Sponsor:** Academic

**Publication Year:**  1995

**Design:**  double-blind placebo controlled safety and efficacy

**Phase:**  I-II

**Treatment duration:** 6 months

**Randomization:**  unknown

**Arms:**  Placebo

BDNF

**Patients/arm (total):** unknown

**Primary endpoint:** unknown

**Secondary endpoints:** FVC (Forced vital capacity)

Walking speed

Additional endpoints unknown

**Results**

**Primary endpoint:**

Unknown

**Secondary endpoints:**

**FVC:** (mean change from baseline FVC% adjusted for baseline value: placebo, -21.3%; BDNF, -14.7%; **p < 0.05**, ANCOVA),

**Walking speed**: A significant slowing of the decline in walking speed (mean change from baseline walking speed in meters per second, adjusted for center and baseline value: placebo, -0.41; BDNF, -0.29; **p < 0.05**, ANCOVA).

**Conclusion**

The study appears to have shown statistically significant benefits on two functional outcomes in ALS patients. The original conference abstract is no longer available from the publisher, therefore no additional conclusions can be dra­­wn. A large-scale Phase III study was launched on the basis of these results.

## BDNF Study Group PIII (Kasarskis et al., 1999)

**Compound:** r-metHuBDNF

**Sponsor:** Amgen

**Publication Year:**  1999

**Design:**  double-blind placebo controlled

**Phase:**  III

**Treatment duration:** 9 months

**Randomization:**  1:1:1

**Arms:**  Placebo

BDNF 25 µg/kg/day

BDNF 100 µg/kg/day

**Patients/arm (total):**  387/374/374 (1135)

**Primary endpoints:** FVC 6 months (forced vital capacity)

Survival 9 months

Alpha **FVC**: 0.013; **Survival:** 0.025

**Secondary endpoints:** ALSFRS

Ashworth Spasticity

FVC 9 months

PATA (syllable repetition)

Respiratory events

SIP-PDS (sickness impact profile score)

Walking speed

**Results**

**Primary endpoints:** no significance on either endpoint

**Secondary endpoints:** no significance on all endpoints

**Conclusion**

Phase III study failed on the primary endpoint. No statistical significance was reached in any of the secondary endpoints.

**Post-hoc analysis**

In the protocol-mandated subset analysis of patients who met all inclusion criteria and who received at least 90% of their assigned dose during the study for the first 2 months, there was a statistically significant survival advantage for patients in the high-dose BDNF group (survival probability of 89.7% versus 85.4%; **p = 0.04**).

There was a significantly greater survival (**p = 0.0001**) among the 78 BDNF patients with diarrhea (during first 15 days of treatment) (97.5%) compared with placebo patients (84.6%) or high-dose BDNF patients who did not have diarrhea (85.8%).

In patients who had an FVC% at baseline of <=91% (representing the lower 60% of baseline FVC values), BDNF showed a dose-related survival effect that was significant for the 100 µg/kg/day group alone versus placebo (**p = 0.03**). Patients who entered the study with baseline serum chloride values of <=100 mEq/L showed a striking survival advantage of BDNF (P values not reported).

# Ceftriaxone

## NCT00349622 – Stage I-II (Berry et al., 2013)

**Compound:** Ceftriaxone

**Sponsor:** NINDS

**Publication Year:**  2013

**Design:**  double-blind placebo controlled safety and tolerability

**Phase:**  I-II

**Treatment duration:** Phase I: 7 days; Phase II: 20 weeks

**Randomization:**  1:1:1

**Arms:**  Placebo (i.v.)

Ceftriaxone 2 g/day (i.v. twice daily)

Ceftriaxone 4 g/day (i.v. twice daily)

**Patients/arm (total):**  21/23/22 (66)

**Primary endpoint:** Phase I: PK data; Phase II: safety and tolerability

**Secondary endpoints:** ALSFRS-R

Vital Capacity

Hand-held dynamometry

**Results**

**Primary endpoint:**

**Phase I:** Drug concentration in the CSF reached pre-specified target levels

**Phase II:** Safety and tolerability was deemed acceptable

**Secondary endpoints:**

Subjects were not unblinded before proceeding to a Phase III trial. No data analysis was possible.

**Conclusion**

This study employed a novel Phase I-III non-stop design, whereby the patients were continuously treated with the study drug in all phases. Phases I-II did not measure functional outcomes. Based on the safety and tolerability data, DSMB recommended to progress directly to a large-scale Phase III trial using the higher dose of ceftriaxone (4 g/day).

## NCT00349622 – Stage III (Cudkowicz et al., 2013b)

**Compound:** Ceftriaxone

**Sponsor:** NINDS

**Publication Year:**  2013

**Design:**  double-blind placebo controlled

**Phase:**  III

**Treatment duration:** 12 months

**Randomization:**  1:2

**Arms:**  Placebo (i.v.)

Ceftriaxone 4g/day (i.v. twice daily)

**Patients/arm (total):** ??/ ?? (513)

**Primary endpoints:** TTD (time to death **or** tracheostomy **or** PAV)

ALSFRS-R

Alpha unknown

**Secondary endpoints:** SVC (slow vital capacity)

HHD (hand-held dynamometry) – upper extremities

HHD (hand-held dynamometry) – lower extremities

ALSQOL (quality of life)

**Results**

**Primary endpoints:**

**TTD: p = 0.3680**

**ALSFRS-R: p = 0.1715**

**Secondary endpoints:**

Not reported

**Conclusion**

Phase III trial (part of a non-stop Phase I-III trial design) failed on the primary endpoints. Study results were presented as a Conference abstract only. No additional information is available.

# Celexocib

## NEALS (Celexocib) (Cudkowicz et al., 2006)

**Compound:** Celexocib

**Sponsor:** Pharmacia/Pfizer

**Publication Year:**  2006

**Design:**  double-blind placebo controlled

**Phase:**  II-III

**Treatment duration:** 12 months

**Randomization:**  1:2

**Arms:**  Placebo

Celexocib 800 mg/day

**Patients/arm (total):** 99/201 (300)

**Primary endpoint:** MVIC 8 arm muscles (maximum voluntary isometric contraction)

Alpha = 0.05

**Secondary endpoints:** ALSFRS-R

CSF PGE2 levels

MUNE (motor unit number estimates)

MVIC 10 leg muscles

Survival

Vital capacity

**Results**

**Primary endpoint:** no significance on primary endpoint

**Secondary endpoints:** no significance on all endpoints

**Conclusion**

This Phase II-III study was launched on the basis of positive preclinical data. No prior Phase II was performed. Riluzole was allowed, and over 65% of all patients were taking it. Study failed on the primary endpoint, no statistical significance was reached in any of the secondary endpoints. Safety and tolerability were acceptable.

# rhCNTF

## ALS CNTF Study Group (ALS CNTF Treatment Study Group, 1996)

**Compound:** rhCNTF

**Sponsor:** Regeneron Pharmaceuticals Inc.

**Publication Year:**  1996

**Design:**  double-blind placebo controlled

**Phase:**  II-III

**Treatment duration:** 9 months

**Randomization:**  1:1:1

**Arms:**  Placebo

rhCNTF **15 µg/kg** // **30 µg /kg**; (s.c. 3x per week)

**Patients/arm (total):** 245/244/241 (730)

**Primary endpoint:** MVC (maximum voluntary isometric contraction - combined megascore)

Alpha = 0.05

**Secondary endpoints:** ALSFRS

FVC (forced vital capacity)

PIF (peak inspiratory flow)

Walking speed

Purdue pegboard test

Oral-labial-lingual dexterity

S&E (modified Schwab and England Scale)

GCIC (Global Clinical Impression of Change)

**Results**

**Primary endpoint:** MVC (Combined Megascore)**: p=0.08** (see Table)

**Secondary endpoints:** no significance on any endpoints (see Table):


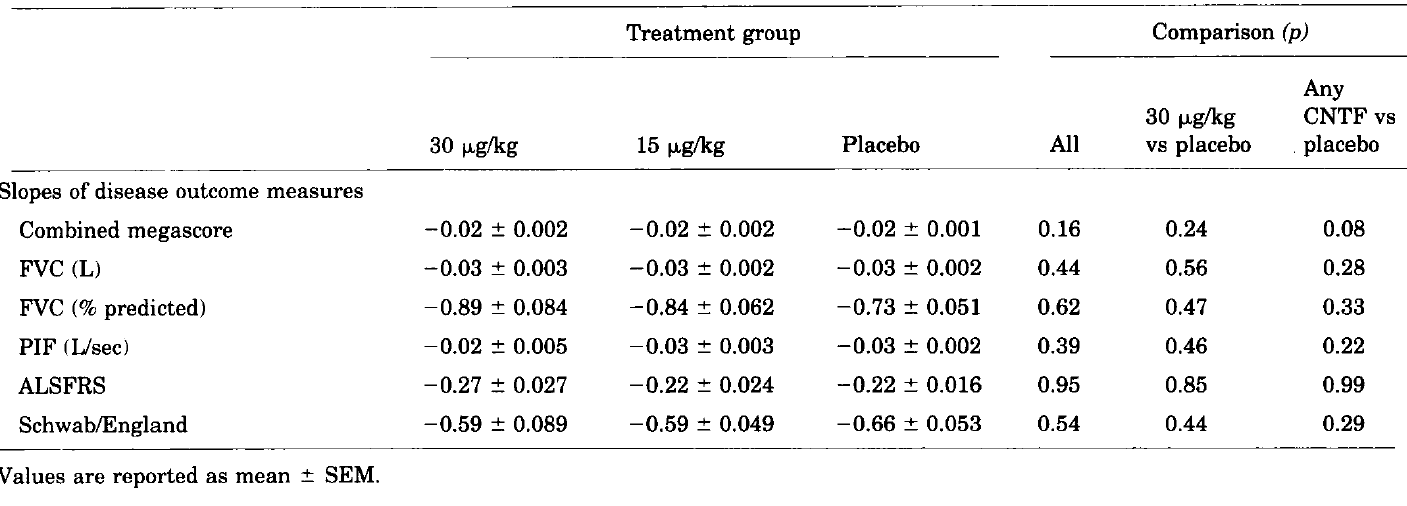


**Conclusion**

This Phase II-III study was launched on the basis of positive preclinical data and the results of the Phase I-II study(ALS CNTF Treatment Study Group, 1995), which demonstrated acceptable safety and tolerability of rhCNTF administration in 57 ALS patients. Phase I-II pre-trial did not measure functional outcomes. This Phase II-III study failed on the primary endpoint. No statistical significance was reached in any of the secondary endpoints.

## rhCNTF Study Group (Miller et al., 1996)

**Compound:** rhCNTF

**Sponsor:** Syntex-Synergen Neuroscience Joint Venture

**Publication Year:**  1996

**Design:**  double-blind placebo controlled

**Phase:**  III

**Treatment duration:** 6 months

**Randomization:**  1:1:1:1

**Arms:**  Placebo

rhCNTF **0.5 µg/kg** // **2.0 µg /kg** // **5.0 µg/kg**; (s.c. daily)

**Patients/arm (total):** 123/124/123/113 (570)

**Primary endpoint:** MVC (maximum voluntary isometric contraction - combined megascore)

Alpha = 0.05

**Secondary endpoints:** Arm megascore (MVC 10-arm muscles)

Leg megascore (MVC 8-leg muscles)

FVC (forced vital capacity)

SIP (sickness impact profile – quality of life)

Survival

**Results**

**Primary endpoint:** no significance on primary endpoint

**Secondary endpoints:** no significance on any of the secondary endpoints

**Conclusion**

This Phase III study was launched on the basis of positive preclinical data and the results of the Phase I study(Miller et al., 1993), which demonstrated acceptable safety and tolerability of rhCNTF administration in ALS patients. Phase I pre-trial did not measure functional outcomes. This Phase III study failed on the primary endpoint. No statistical significance was reached in any of the secondary endpoints.

# Coenzyme Q10

## Ferrante 2005 (Kaufmann et al., 2009)

**Compound:** Coenzyme Q10

**Sponsor:** Muscular Dystrophy Association

**Publication Year:**  2005

**Design:**  open-label dose-escalation

**Phase:**  IIa

**Treatment duration:** 8 months

**Randomization:**  not applicable

**Arms:**  Coenzyme Q10 dose escalation (600 – 3000 mg/day)

**Patients/arm (total):** 31

**Primary endpoint:** Safety and tolerability

**Safety endpoints*:** ALSFRS-R

MVIC (maximum voluntary isometric contraction)

VC (vital capacity)

Survival

*****Control group was taken from a placebo group in another ALS clinical trial (topiramate). Functional outcomes were considered safety measures and no efficacy assessments were carried out. These were included to ensure that the study drug treatment does not lead to the worsening of functional parameters.

**Results**

**Primary endpoint:** Coenzyme Q10 was safe and well tolerated in 31 subjects treated with doses as high as 3,000 mg/day for 8 months

**Safety endpoints:** No worsening of functional outcomes was observed across the whole dosage range

**Conclusion**

Coenzyme Q10 is safe and well tolerated in ALS patients. While plasma Coenzyme Q10 levels increased with each dose escalation in this study, there was no statistical difference in plasma levels between the 2,400 and the 3,000 mg/day groups.

## NCT00243932 – Stage 1 (Kaufmann et al., 2009)

**Compound:** Coenzyme Q10

**Sponsor:** NINDS

**Publication Year:**  2009

**Design:**  adaptive, 2-stage double-blind placebo controlled

**Phase:**  II

**Treatment duration:** 9 months

**Randomization:**  1:1:1

**Arms:**  Placebo

Coenzyme Q10 (**1800 mg/day** // **2700 mg/day**)

**Patients/arm (total):** 35:35:35 (105)

**Primary endpoint:** ALSFRS-R

Alpha = 0.10

**Secondary endpoints:** None

The study utilized a multi-center, randomized, stratified, placebo-controlled, double-blind, two-stage, adaptive, bias-corrected, intent-to-treat (ITT) design. **Stage 1** selected one of two doses of Coenzyme Q10 (1,800 or 2,700 mg daily) based on an initial sample comparison. **Stage 2** assessed whether the preferred dose shows sufficient promise to warrant Phase III testing.

**Results**

**Primary endpoint:** In December 2006 the Clinical Principal Investigator was given results by treatment group without identifying which group was which. The results were prepared by the study statistician and shared with the DSMB before they were provided to the clinical principal investigator. The investigators proposed, and the DSMB and NINDS accepted after data review, that in the absence of safety concerns, the group with the lower ALSFRS-R decline should be selected (**2,700 mg/day**), per the **Stage 1** design.

**Conclusion**

The 35 participants who had received the **2,700** mg/day dose in **Stage 1**, and the 35 concurrent placebo controls, were carried forward for inclusion in the bias-corrected **Stage 2** futility analysis. The 80 additional patients needed for stage 2 (40 at Coenzyme Q10 2,700 mg/day, and 40 placebo controls) were randomized between February and May, 2007. Stage 2 efficacy results are presented in the following section.

## NCT00243932 – Stage 2 (Kaufmann et al., 2009)

**Compound:** Coenzyme Q10

**Sponsor:** NINDS

**Publication Year:**  2009

**Design:**  adaptive, 2-stage double-blind placebo controlled

**Phase:**  II

**Treatment duration:** 9 months

**Randomization:**  1:1

**Arms:**  Placebo

Coenzyme Q10 (2700 mg/day)

**Patients/arm (total):** 75/75 (150)

**Primary endpoint:** ALSFRS-R

Alpha = 0.10

**Secondary endpoints:** FVC (forced vital capacity)

FSS (fatigue severity scale)

SF36 Health Survey (quality of life)

**Results**

**Primary endpoint:** not significant: **p=0.14** (See table)

**Secondary endpoints:** no significance on any endpoints (see table)


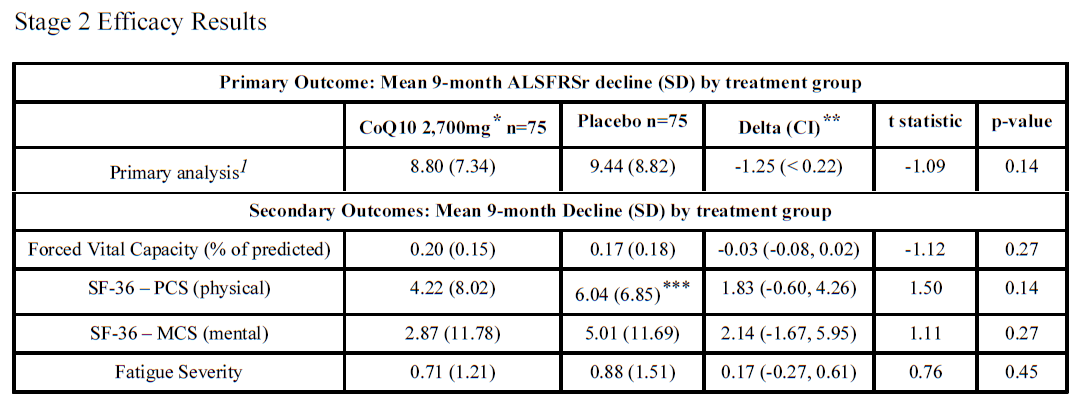


**Conclusion**

The study failed on the primary endpoint. No statistical significance was reached in any of the secondary endpoints. The study was thus terminated for futility. Proceeding to a larger scale Phase III trial was not recommended.

# Creatine

## Groeneveld 2003 (Groeneveld et al., 2003)

**Compound:** Creatine monohydrate

**Sponsor:** Academic

**Publication Year:**  2003

**Design:**  sequential double-blind placebo controlled

**Phase:**  II-III

**Treatment duration:** 16 months

**Randomization:**  1:1

**Arms:**  Placebo

Creatine 10 gr/day

**Patients/arm (total):** 87/88 (175)

**Primary endpoint:** DTP (time to death **or** tracheostomy **or** PAV)

Alpha = 0.05

**Secondary endpoints:** ALSFRS

FVC (forced vital capacity)

MVIC 8 arm muscles (maximum voluntary isometric contraction)

SF-36 QOL (quality of life)

**Results**

**Primary endpoint:** no significance on primary endpoint

**Secondary endpoints:** no significance on all endpoints

**Conclusion**

The study was launched on the basis of positive preclinical data. Phase II-III study failed on the primary endpoint. No statistical significance was reached in any of the secondary endpoints.

## NCT00005674 (NEALS) (Pastula et al., 2012; Shefner et al., 2004)

**Compound:** Creatine monohydrate

**Sponsor:** NCRR

**Publication Year:**  2004

**Design:**  double-blind placebo controlled

**Phase:**  II

**Treatment duration:** 6 months

**Randomization:**  1:1

**Arms:**  Placebo

Creatine 5 gr/day

**Patients/arm (total):** 52/52 (104)

**Primary endpoint:** MVIC 8 arm muscles (maximum voluntary isometric contraction)

Alpha = 0.05

**Secondary endpoints:** ALSFRS-R

Grip strength

MUNE (motor unit number estimates)

Survival

**Results**

**Primary endpoint:** no significance on primary endpoint

**Secondary endpoints:** no significance on all endpoints

**Conclusion**

The study was launched on the basis of positive preclinical data. Phase II study failed on the primary endpoint. No statistical significance was reached in any of the secondary endpoints.

## NCT00069186 (Pastula et al., 2012; Rosenfeld, 2001; Rosenfeld et al., 2008)

**Compound:** Creatine monohydrate

**Sponsor:** Academic/ Avicena Group

**Publication Year:**  2008

**Design:**  double-blind placebo controlled

**Phase:**  III

**Treatment duration:** 9 months

**Randomization:**  1:1

**Arms:**  Placebo

Creatine 5 gr/day

**Patients/arm (total):** 54/53 (107)

**Primary endpoint:** MVIC 10 upper muscles (maximum voluntary isometric contraction)

Alpha = 0.05

**Secondary endpoints:** ALSFRS-R

FVC (forced vital capacity)

Muscle fatigue

SF-12 (quality of life)

**Results**

**Primary endpoint:** no significance on primary endpoint

**Secondary endpoints:** no significance on all endpoints

**Conclusion**

Phase III study failed on the primary endpoint. No statistical significance was reached in any of the secondary endpoints.

Authors launched this study on the basis of the apparently statistically significant results of a pilot Phase I-II study. The results of which were presented at the American Academy of Neurology Annual Meeting (2001), abstract for which is no longer available at the publishers website. The extract from the 2008 Phase III results publication follows:

“Prior to the onset of this study, a nine-month placebo controlled, pilot study of creatine monohydrate in 21 patients with ALS showed either a significantly greater improvement in strength as measured by maximal voluntary isometric contraction (MVIC) or a more modest decline compared to patients taking placebo. The most profound effects were noted at the earliest time point(s), within the first month. The pilot study also showed a trend favoring an improvement in FVC associated with improved MVIC in the creatine group.”

# Dexpramipexole

## NCT00647296 – Part 1 (Cudkowicz et al., 2011)

**Compound:** Dexpramipexole

**Sponsor:** Biogen

**Publication year:**  2011

**Design:**  double-blind placebo controlled safety and tolerability

**Phase:**  II

**Treatment duration:** 12 weeks

**Randomization:**  1:1:1:1

**Arms:**  Placebo

Dexpramipexole 50, 150, 300 mg/day

**Patients/arm (total):** 27/23/26/26 (102)

**Primary assessment endpoint:** Safety

**Secondary endpoints:** ALSFRS-R

McGill SIS (single-item scale QoL)

Survival

Vital Capacity

**Results**

**Primary endpoint:** Acceptable safety and tolerability

**Secondary endpoints:**

**ALSFRS-R**: The primary analysis of overall treatment effect by the linear mixed-effects model applied to the slope was not significant (**P = 0.11**). When a six-point or greater drop in ALSFRS-R total score from baseline to 12 weeks was used to define subjects that failed to respond to drug treatment, a significant dose-dependent effect was observed. The number of failures totaled nine subjects (33%) in the placebo group, eight subjects (35%) in the 50-mg group, four subjects (15%) in the 150-mg group and two subjects (8%) in the 300-mg group (logistic regression analysis, **P=0.01**).

**McGill SIS (single-item scale QoL):** not significant

**Survival:** not applicable (applicable for Part 2 of the study)

**Vital Capacity slope:** linear mixed-effects model (**P=0.54**); logistic regression on dosage (treatment failure defined as >20% drop in VC from baseline to week 12) (**P=0.03**)

**Conclusion**

Part 1 of this 2-part study had demonstrated acceptable safety for dexpramipexole. No statistical significance was reached in functional outcomes in any of the secondary endpoints according to the primary statistical analysis.

## NCT00647296 – Part 2 (Cudkowicz et al., 2011)

**Compound:** Dexpramipexole

**Sponsor:** Biogen

**Publication Year:**  2011

**Design:**  double-blind active controlled safety and tolerability

**Phase:**  II

**Treatment duration:** 24 weeks

**Randomization:**  1:1

**Arms:**  Dexpramipexole 50, 300 mg/day

**Patients/arm (total):** 48/44 (92)

**Primary assessment endpoint:** Safety

**Secondary endpoints:** ALSFRS-R

McGill SIS (single-item scale QoL)

Survival

Vital Capacity

CAFS

**Results**

**Primary endpoint:** Acceptable safety and tolerability

**Secondary endpoints:**

**ALSFRS-R** **slope**: estimates (-1.28 (50 mg), -1.02 (300 mg)) this is 20.5% attenuation . **P=0.177**

**McGill SIS (single-item scale QoL):** not significant

**Survival:** Hazard of mortality: 68% reduction 300-mg group relative to the 50-mg group (log-rank test, **P = 0.07**; hazard ratio 0.32, 95% CI: 0.086 to 1.18, Kaplan-Meier estimates)

**Vital Capacity:** not significant

**CAFS Joint Rank Analysis (both ALSFRS-R and mortality combined): P=0.046**

**Conclusion**

Part 2 of this 2-part study had demonstrated acceptable safety for dexpramipexole. In functional assessments, statistical significance was reached for one endpoint only, with CAFS Joint Rank Analysis demonstrating treatment effect. A large-scale Phase III study was launched on this basis.

## NCT01281189 (EMPOWER) (Bozik et al., 2014; Cudkowicz et al., 2013a)

**Compound:** Dexpramipexole

**Sponsor:** Biogen

**Publication Year:**  2013

**Design:**  double-blind placebo controlled

**Phase:**  III

**Treatment duration:** 12 months

**Randomization:**  1:1

**Arms:**  Placebo

Dexpramipexole 300 mg/day

**Patients/arm (total):** 468/474 (943)

**Primary endpoint:** CAFS

Alpha = 0.05

**Secondary endpoints:** ALSAQ-5

DRI (tracheostomy or non-invasive ventilation >22 hours)

Time to Death

Respiratory decline

SVC (slow vital capacity)

HHD megascore (handheld dynamometry)

**Results**

**Primary endpoint:**

Failure: Least-square mean CAFS scores at 12 months did not differ between participants in the dexpramipexole group (score 441·76, 95% CI 415·43–468·08) and those in the placebo group (438·84, 412·81–464·88; **P=0.86**).

**Secondary endpoints:** no significance on all endpoints

**Conclusion**

Phase III study failed on the primary endpoint. No statistical significance was reached in any of the secondary endpoints. Drug development for ALS was terminated in 2013.

**Post-hoc analysis**

Post-hoc analysis found a positive treatment effect in a subset of patients only. Separation of the treated subgroup to patients with definite El Escorial criteria ALS, with concomitant riluzole and short symptom duration:

- ALSFRS-R: reduced slope decline (0.49, 95% CI 0.10 – 0.87; **p=0.015**)
- Decreased mortality: (H.R. 0.37, 95% CI 0.17 – 0.80; **p=0.011**)
- Reduction in creatinine loss: (4.8 μ m/l; 95% CI 1.6 – 8.0, **p=0.003**)

# Edaravone

## Yoshino 2006 (Yoshino and Kimura, 2006)

**Compound:** Edaravone

**Sponsor:** Mitsubishi Tanabe Pharma Corporation

**Publication Year:**  2006

**Design:**  uncontrolled open-label

**Phase:**  II

**Treatment duration:** 6 months

**Randomization:**  unspecified

**Arms:**  Edaravone 30 mg (daily IV injections, two weeks on, two weeks off)

Edaravone 60 mg (daily IV injections, two weeks on, two weeks off)

**Patients/arm (total):** 5/14 (19)

**Primary endpoint:** ALSFRS-R

Alpha = 0.05

**Secondary endpoints:** Muscle function

Respiratory function

Blood gases

CSF protein (total protein, Alb, IgG) and 3NT

lipid peroxide levels in the CSF and blood

**Results**

Statistical analysis was performed with Wilcoxon signed rank test, comparing the functional decline observed in the 6 months treatment period versus functional decline in the 6 months **prior** to treatment.

**Primary endpoint:** 30 mg (**p= 0.5**), 60 mg (**p= 0.039**)

**Secondary endpoints:**

**CSF 3NT protein levels**: marked reduction in the 60 mg group (no p value specified)

**All other secondary endpoints:** Not discussed

**Conclusion**

This exploratory Phase II study was successful in the primary endpoint in the 60 mg dose group. However, this was a small scale uncontrolled open label study and the comparisons were made in functional decline between the 6 months period prior to treatment versus the 6 months of drug treatment. A confirmatory Phase II study was subsequently launched

## NCT00330681 (Abe et al., 2014)

**Compound:** Edaravone

**Sponsor:** Mitsubishi Tanabe Pharma Corporation

**Publication Year:**  2014

**Design:**  double-blind placebo controlled

**Phase:**  III

**Treatment duration:** 36 weeks (12 weeks observation + 24 weeks treatment)

**Randomization:**  1:1

**Arms:**  Edaravone 60 mg (daily IV injections, two weeks on, two weeks off)

**Patients/arm (total):** 104/102 (206)

**Primary endpoint:** ALSFRS-R

Alpha = 0.05

**Secondary endpoints:** FVC (forced vital capacity)

Grip strength (left/right mean)

Pinch strength (left/right mean)

Norris Scale (modified)

ALSAQ-40 (quality of life)

**Results**

**Primary endpoint:** not significant (see table)

**Secondary endpoints:** no significance on all endpoints (see table)

**
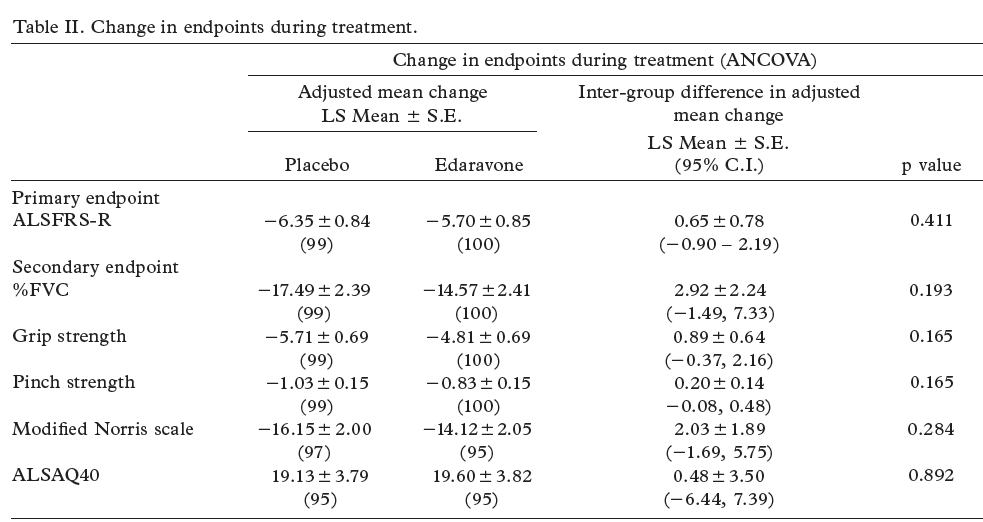
**

**Conclusion**

This confirmatory Phase III study failed on all endpoints. Two subsequent confirmatory Phase III trials with narrowed inclusion criteria were subsequently launched. The results of the confirmatory trials were published as conference abstracts only (Tanaka et al., 2016a, 2016b) and had not been published in peer-reviewed sources.

# Erythpoietin

## EudraCT # 2005-005873-31 (Lauria et al., 2009)

**Compound:** Erythropoietin + riluzole

**Sponsor:** National Neurological Institute (Milan)

**Publication Year:**  2009

**Design:**  double-blind placebo controlled

**Phase:**  II

**Treatment duration:** 6-month lead-in + 24 months treatment

**Randomization:**  1:1

**Arms:**  Placebo

IV Erythropoietin (40 000 IU) once/3 weeks (first 3m)//once/2 weeks till end

**Patients/arm (total):** 11/12 (23)

**Primary endpoints:** Adverse events and safety

Death **or** tracheotomy

Alpha = 0.05

**Secondary endpoint:** ALSFRS-R

**Results**

**Primary endpoints:** Acceptable safety and tolerability

**Death or tracheotomy**: not significant (see Figure)


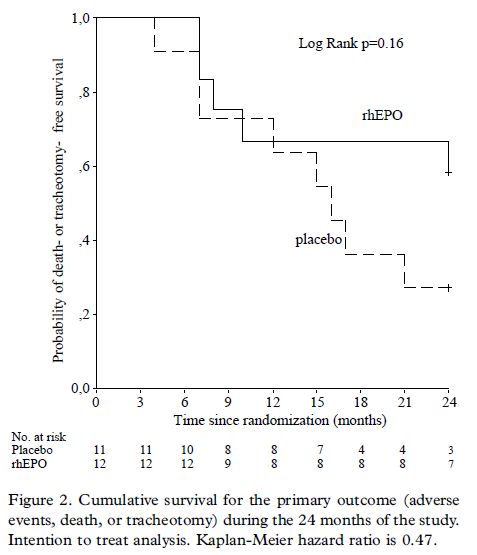


**Secondary endpoint:** Not significant

**Conclusion**

Phase II study failed on the primary efficacy endpoint. No statistical significance was reached in the secondary endpoint. Study was not powered to reach significance on efficacy. Phase III study was launched on the basis of these results.

## EudraCT # 2009-016066-91 (Lauria et al., 2015)

**Compound:** Erythropoietin + riluzole

**Sponsor:** National Neurological Institute (Milan)

**Publication Year:**  2015

**Design:**  double-blind placebo controlled

**Phase:**  III

**Treatment duration:** 12 months

**Randomization:**  1:1

**Arms:**  Placebo

IV Erythropoietin (40 000 IU) once/2 weeks

**Patients/arm (total):** 97/103 (200)

**Primary endpoint:** time to death **or** tracheotomy **or** NIV (non-invasive ventilation)

Alpha = 0.05

**Secondary endpoints:** ALSFRS-R

SVC (slow vital capacity)

ALSAQ-40 (quality of life)

**Results**

**Primary endpoint:** not significant

**Secondary endpoints:** no significance on all endpoints

**Conclusion**

Phase III study failed on the primary endpoint. No statistical significance was reached in any of the secondary endpoints.

# Glatiramer acetate

## Gordon 2006 (Gordon et al., 2006)

**Compound:** Glatiramer acetate

**Sponsor:** Teva pharmaceuticals

**Publication Year:**  2006

**Design:**  randomized controlled*

**Phase:**  II

**Treatment duration:** 6 months

**Randomization:**  1:1:1

**Arms:**  Control*

Glatiramer acetate 20 mg s.c. (1xday)

Glatiramer acetate 20 mg s.c. (1x every 2 weeks)

**Patients/arm (total):** 10/10/10 (30)

**Primary endpoint:** Safety and tolerability

**Secondary endpoints:** Immunogenicity (T cell proliferation)

*Control group was composed of blinded patients from another clinical trial (minocycline). As Glatiramer acetate is essentially a « vaccine », the purpose of the control group was to demonstrate the absence of immune reactivity (therefore, authors considered irrelevant the fact that control group was composed of blinded placebo **or** minocycline-treated patients).

**Results**

**Primary endpoint:** Acceptable safety and tolerability

**Secondary endpoint:** T cell proliferation changes with time in both GA-treated groups (**p=0.02**). The changes were most significant with less frequent dosing (1x every 2 weeks) (**p=0.01**).

**Conclusion**

The purpose of the study was to determine safety and tolerability of GA vaccination in ALS patients. The hypothesis behind this was that immunomodulation may improve functional outcomes in ALS patients (as demonstrated in preclinical mouse models). The functional outcomes were not tested in this study (only T cell proliferation was tested). On the basis of the results of this Phase I-II study and preclinical data, a large-scale Phase II-III was launched.

## EudraCT (2006-001688-49) (Meininger et al., 2009)

**Compound:** Glatiramer acetate

**Sponsor:** Teva pharmaceuticals

**Publication Year:**  2009

**Design:**  double-blind placebo controlled

**Phase:**  II-III

**Treatment duration:** 52 weeks

**Randomization:**  1:1

**Arms:**  Placebo

Glatiramer acetate 40 mg s.c. (1xday)

**Patients/arm (total):** 182/184 (366)

**Primary endpoint:** ALSFRS-R

Alpha = 0.05

**Secondary endpoint:** DTP (time **to** death **or** tracheostomy or PAV)

**Results**

**Primary endpoint: ALSFRS-R:** not significant (**p=0.48**)

**Secondary endpoint:** not significant (**p=0.75**)

**Conclusion**

Phase II-III study failed on the primary endpoint. No statistical significance was reached in the secondary endpoint.

# r-Hu-IGF-1

## Lai 1997 (Lai et al., 1997)

**Compound:** recombinant human IGF-I

**Sponsor:** Cephalon/Chiron Corp/Muscular Dystrophy Association

**Publication Year:**  1997

**Design:**  double-blind placebo controlled

**Phase:**  III

**Treatment duration:** 9 months

**Randomization:**  1:1:1

**Arms:**  Placebo

IGF-1 s.c. (0.05 mg /kg/day)

IGF-1 s.c. (0.10 mg /kg/day)

**Patients/arm (total):** 90/89/87 (266)

**Primary endpoint:** AALS (Appel ALS rating scale) /baseline to end of treatment

Alpha = 0.05

**Secondary endpoints:** AALS total score changes (from baseline by month)

Time to prespecified termination criteria (AALS ≥115 **or** FVC <39%)

SIP (sickness impact profile)

**Results**

**Primary endpoint: AALS:** both doses combined – failure (**p=0.054**)

*26% slower rate of symptoms progression in high-dose group versus placebo (**p=0.01**)

**Secondary endpoints:**

**AALS score change:** Statistical significance was reached in high-dose group only, starting at month 2 (**p<0.05**)

**Time to AALS ≥115 or FVC <39%:** The relative risk of a protocol-specified early termination was 0.56 (95% CI, 0.32, 0.97) for the high-dose group as compared with the placebo group (**p = 0.04**), indicating that high-dose patients had a 44% lower risk of these events. There was no significant difference in risk between the low-dose and placebo patients (RR, 1.02; 95% CI, 0.63, 1.65).

**SIP:** Dose-related and statistically significant differences between the placebo and the high-dose rhIGF-I-treated groups for the overall (**p = 0.01**) and psychosocial dimension (**p = 0.02**) scores. The mean increase in the physical dimension score was 30% less for the high-dose group than for placebo, although this difference was not statistically significant (**p = 0.08**).

**Conclusion**

The study was launched on the basis of positive preclinical data and the results of a successful Phase I trial, which demonstrated that IGF-1 treatment was safe and well tolerated in ALS patients.

This Phase III study failed on the primary endpoint. Statistical significance was reached in high-dose treated patients only, both in the primary and secondary endpoints. Authors acknowledge that the treatment effect was very modest, and that the results of the study were inconclusive, warranting additional Phase III trials.

## Borasio 1998 (Borasio et al., 1998)

**Compound:** recombinant human IGF-I

**Sponsor:** Cephalon/Chiron Corp

**Publication Year:**  1998

**Design:**  double-blind placebo controlled

**Phase:**  III

**Treatment duration:** 9 months

**Randomization:**  1:2

**Arms:**  Placebo

IGF-1 s.c. (0.10 mg /kg/day)

**Patients/arm (total):** 59/124 (183)

**Primary endpoint:** AALS (Appel ALS rating scale) /baseline to end of treatment

Alpha = 0.05

**Secondary endpoint:** SIP (sickness impact profile)

**Results**

**Primary endpoint: AALS:** The mean change in AALS total score from baseline of the patients treated with IGF-I was 21.9 ± 1.5 versus 25.2 ± 2.3 for the placebo group (δ = -3.3 points; 95%CI -8.7, 2.0; **p = 0.22**)

**Secondary endpoint:**

**SIP:** No significant difference between treatment groups was found in the SIP scores (change from baseline: IGF-I, 9.4 ± 1.1; placebo, 10.5 ± 1.6; **p = 0.59**).

**Conclusion**

The study was launched on the basis of positive preclinical data and the results of a successful Phase I trial, which demonstrated that IGF-1 treatment was safe and well tolerated in ALS patients.

This Phase III study failed on the primary endpoint. No statistical significance was reached in the secondary endpoint.

## NCT00035815 (Sorenson et al., 2008)

**Compound:** recombinant human IGF-I

**Sponsor:** NIH / ALS Association

**Publication Year:**  2008

**Design:**  double-blind placebo controlled

**Phase:**  III

**Treatment duration:** 2 years

**Randomization:**  1:1

**Arms:**  Placebo

IGF-1 s.c. (0.05 mg /kg/ twice daily)

**Patients/arm (total):** 163/167 (330)

**Primary endpoint:** MMT (manual muscle testing score)

Alpha = 0.05

**Secondary endpoints:** ALSFRS-R

Tracheostomy-free survival

**Results**

**Primary endpoint: MMT:** There was an overall mean rate of change in the MMT score of 0.41 units per month. The IGF-1 treatment group changed at a rate of 0.44 units per month and the placebo group changed at a rate of 0.39 units per month (**p=0.529**).

**Secondary endpoints:**

**ALSFRS-R**: The overall mean rate of change for the ALSFRS-r was 2.35 units per month. The IGF-I treatment group changed at a rate of 2.5 units per month and the placebo group changed at a rate of 2.2 units per month (**p= 0.321**).

**Tracheostomy-free survival**: not significant

**Conclusion**

The study was launched on the basis of the inconsistent results reported in 2 previous Phase III trials.

This Phase III study failed on the primary endpoint. No statistical significance was reached in any of the secondary endpoints.

# Lithium

## Fornai 2008 (Fornai et al., 2008)

**Compound:** Lithium carbonate

**Sponsor:** Academic

**Publication Year:**  2008

**Design:**  open-label parallel-group randomized

**Phase:**  II

**Treatment duration:** 15 months

**Randomization:**  unspecified

**Arms:**  riluzole

Lithium carbonate (plasma levels 0.4 to 0.8 mEq/liter) + riluzole

**Patients/arm (total):** 28/16 (44)

**Primary endpoint:** Survival

Alpha = 0.05

**Secondary endpoints:** ALSFRS-R

Norris scale

QoL (SL-36)

Muscle strength (MRC scale)

FVC (forced vital capacity)

**Results**

**Primary endpoint:** No deaths in lithium arm compared with 8 deaths in riluzole group (29%) (**P<0.05**)

**Secondary endpoints:** QOL (SL-36) scale results were not significant. Rest of the endpoints below:


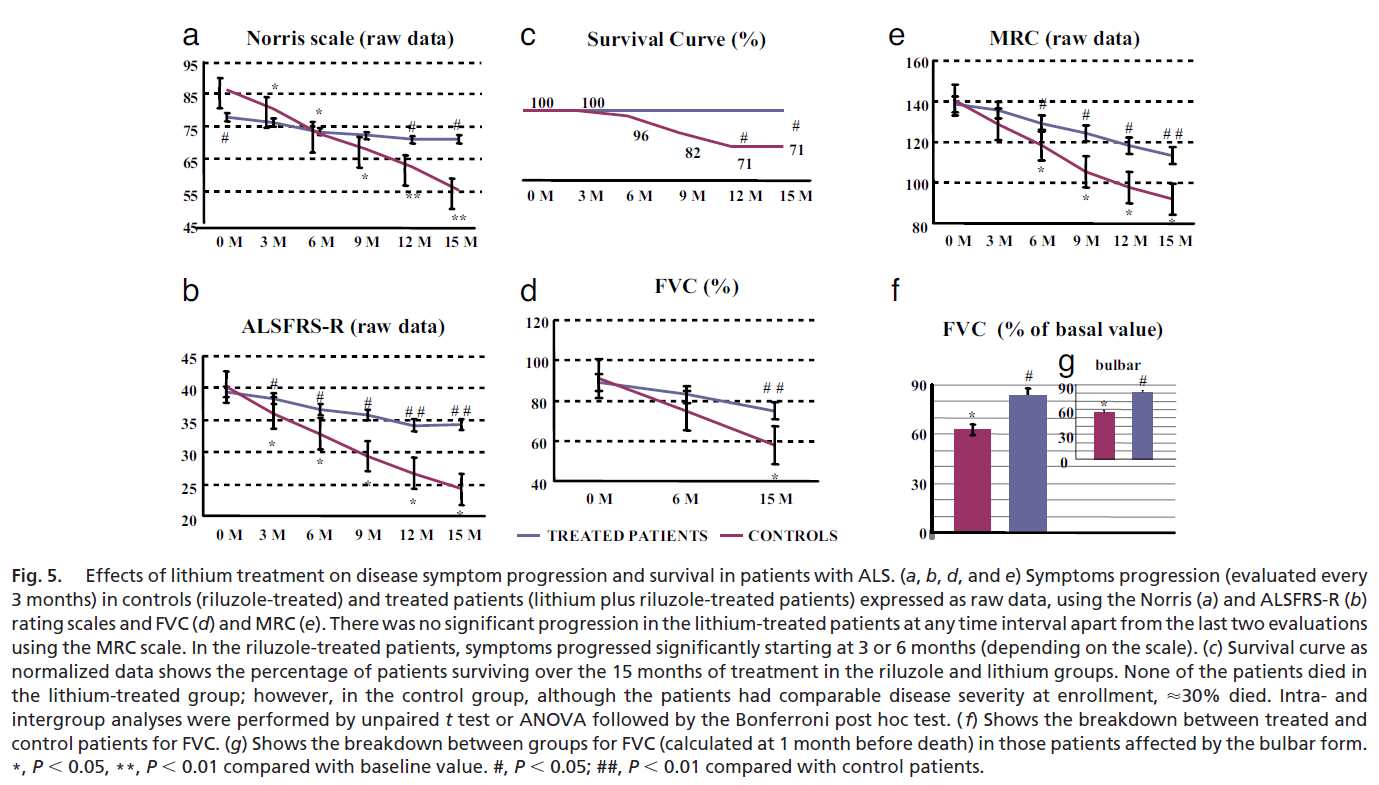


**Conclusion**

Phase II study showed benefits of lithium treatment. The study lacked placebo-control, recruited very few patients and the randomization methods were not clearly described. The study report generated substantial controversy at the time of publication, questioning the validity of the results. A number of follow-up studies were launched on the basis of these results. All subsequent follow-up studies failed.

## NCT00818389 - (NEALS) (Aggarwal et al., 2010)

**Compound:** Lithium carbonate + riluzole

**Sponsor:** Pharmacia, Pfizer

**Publication Year:**  2010

**Design:**  double-blind placebo controlled

**Phase:**  II-III

**Treatment duration:** 9 months

**Randomization:**  1:1

**Arms:**  Placebo

Lithium carbonate (150-1050 mg/day) + riluzole

**Patients/arm (total):** 44/40 (84)*

**Primary endpoint:** ALSFRS-R time to event (decrease of >6 points or death)

Alpha = 0.05

**Secondary endpoints:** ALSFRS-R

ALSSQOL (ALS-specific quality of life)

QIDS-SR (depressive symptomatology self-report)

SVC (slow vital capacity)

Tracheostomy-free survival

*Study initially planned to enroll 250 patients but was stopped for futility at the first interim analysis

**Results**

**Primary endpoint:** not significant

**Secondary endpoints:** no significance on all endpoints

**Conclusion**

Phase III study failed on the primary endpoint. No statistical significance was reached in any of the secondary endpoints. Study was stopped early for futility.

## NTR1448 - (Netherlands) (Verstraete et al., 2012)

**Compound:** Lithium carbonate + riluzole

**Sponsor:** Academic

**Publication Year:**  2012

**Design:**  double-blind placebo controlled

**Phase:**  IIb

**Treatment duration:** 16 months

**Randomization:**  1:1

**Arms:**  Placebo

Lithium carbonate (plasma levels 0.4 to 0.8 mEq/liter) + riluzole

**Patients/arm (total):** 67/66 (133)*

**Primary endpoint:** Survival

Alpha = 0.05

**Secondary endpoints:** ALSFRS-R

FVC (forced vital capacity)

*Study used sequential design, without pre-specified number of patients. It was stopped for futility once 133 patients were recruited

**Results**

**Primary endpoint:** not significant

**Secondary endpoints:** no significance on all endpoints

**Conclusion**

Phase III study failed on the primary endpoint. No statistical significance was reached in any of the secondary endpoints. Study was stopped early for futility.

## LiCALS - EudraCT (2008-006891-31) (Morrison et al., 2013)

**Compound:** Lithium carbonate + riluzole

**Sponsor:** Motor Neuron Disease Association (GB and Ireland)

**Publication Year:**  2013

**Design:**  double-blind placebo controlled

**Phase:**  III

**Treatment duration:** 18 months

**Randomization:**  1:1

**Arms:**  Placebo

Lithium carbonate (300-900 mg /day) + riluzole

**Patients/arm (total):** 107/107 (214)

**Primary endpoint:** Survival

Alpha = 0.05

**Secondary endpoints:** ALSFRS-R

EuroQoL (quality of life)

Mental-health state

**Results**

**Primary endpoint:** not significant

**Secondary endpoints:** no significance on all endpoints

**Conclusion**

Phase III study failed on the primary endpoint. No statistical significance was reached in any of the secondary endpoints

# Masitinib

**Compound:** Masitinib (AB1010) + Riluzole

**Sponsor:** AB Science

**Publication Year:**

**Design:**  double-blind placebo controlled

**Phase:**  II-III

**Treatment duration:** 48 weeks

**Randomization:**  1:1:1

**Arms:**  Placebo

Masitinib 3.0 mg/kg/day

Masitinib 4.5 mg/kg/day

**Patients/arm (total):** 127/127/127 (382)

**Primary endpoint:** ALSFRS-R (baseline to end of treatment)

Alpha = 0.05

**Secondary endpoints:** FVC (forced vital capacity)

CAFS (Combined assessment of function and survival)

ALSAQ-40 (quality of life)

Overall survival

**Results**

Interim analysis was carried out when 50% (191) patients had reached the 48-week treatment period. Interim analysis evaluated ALSFRS-R as the primary endpoint and changes in the FVC, CAFS, ALSAQ-40 and overall survival secondary endpoints.

**Primary endpoint:**

**ALSFRS:** Success: **p <0.01** in the ITT population

**Secondary endpoints:**

The interim analysis showed statistical significance in FVC, CAFS and ALSAQ-40 endpoints and a trend in improvement of overall survival.

**Conclusion**

Masitinib had shown efficacy on both primary and secondary efficacy endpoints in the interim analysis

# Memantine

## NCT00353665 (de Carvalho et al., 2010)

**Compound:** Memantine

**Sponsor:** Academic

**Publication Year:**  2010

**Design:**  double-blind placebo controlled

**Phase:**  II-III

**Treatment duration:** 12 months

**Randomization:**  1:1

**Arms:**  Placebo

Memantine titration in 5 mg/week increments from 5 mg/day to 10 mg/b.i.d.

**Patients/arm (total):** 31/32 (63)

**Primary endpoint:** ALSFRS

Alpha = 0.05

**Secondary endpoints:** FVC (forced vital capacity)

MMT (manual muscle testing – 32 muscle groups – MRC scale)

VAS (visual analogue scales – both patient and medical)

SF-36 (quality of life)

MUNE (motor unit number estimation – both hands)

NI (Neurophysiological index - both hands)

**Results**

**Primary endpoint: ALSFRS:** failure (**p=0.46**); (see table)

**Secondary endpoints:** no significance on all endpoints (see table)


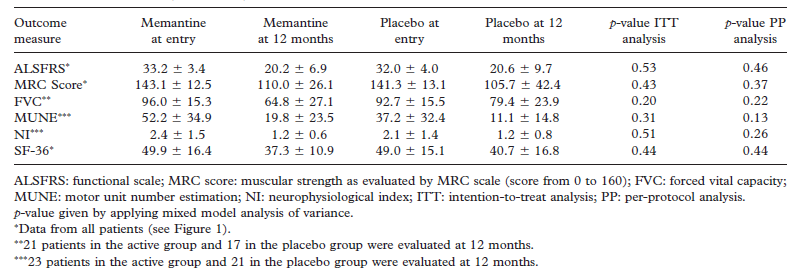


**Conclusion**

The study was launched on the basis of positive preclinical data and the fact that memantine is a moderate-affinity voltage-dependent non-competitive antagonist at glutamatergic NMDA receptors. (Riluzole, the only ALS treatment approved for patients in Europe and the USA, is a possible glutamate antagonist in addition to its proven mechanism of action as a sodium channel blocker).

Phase II-III study failed on the primary endpoint. No statistical significance was reached in any of the secondary endpoints.

# Minocycline

## Gordon 2004 (Gordon et al., 2004)

**Compound:** Minocycline

**Sponsor:** Academic

**Publication Year:**  2004

**Design:**  double-blind placebo controlled feasibility

**Phase:**  I-II

**Treatment duration:** **Trial 1**: 6 months; **Trial 2**: 8 months crossover

**Randomization:**  **Trial 1**: 1: 1; **Trial 2** N/A

**Arms:**  Placebo

**Trial 1**: Minocycline 100 mg / twice daily

**Trial 2:** Minocycline escalating dose (up to a max of 400 mg/ twice daily)

**Patients/arm (total):** **Trial 1**: 9/10 (19)

**Trial 2:** 5/18 (23)

**Primary endpoint:** Safety and tolerability

**Secondary endpoints:** ALSFRS-R

FVC

Grip

MMT

**Results**

**Primary endpoint:** Safety and tolerability acceptable

**Secondary endpoints:** ALSFRS-R: drug treatment effect reached significance in **Trial 2** (**p = 0.047**). Rest of the efficacy measures did not reach statistical significance (see Table below):


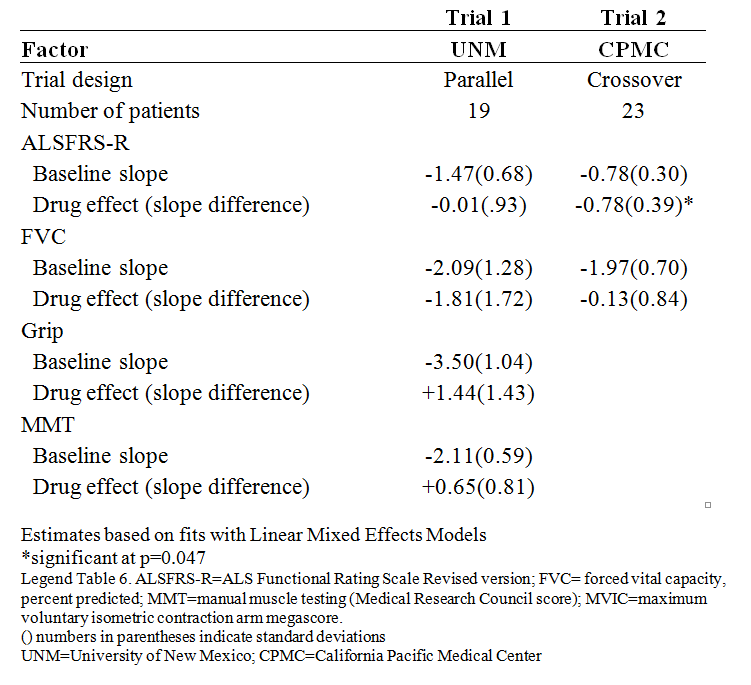


**Conclusion**

These 2 Phase I-II Studies were feasibility studies, designed to determine the safety and tolerability of minocycline, which were found acceptable. Functional outcome measures were also measured. Statistical significance was reached for ALSFRS-R score in Trial 2.

## Pontieri 2005 (Pontieri et al., 2005)

**Compound:** Minocycline

**Sponsor:** Academic

**Publication Year:**  2005

**Design:**  open label active controlled pilot study

**Phase:**  I-II

**Treatment duration:** 6 months

**Randomization:**  1:1

**Arms:**  riluzole

Minocycline 100 mg (1x daily) + riluzole

**Patients/arm (total):** 10/10 (20)

**Primary endpoint:** Safety and tolerability

**Secondary endpoints:** ALSFRS

Complete pulmonary function (no further specification)

**Results**

**Primary endpoint:** Safety and tolerability acceptable

**Secondary endpoints:**

**ALSFRS:** **p = 0.854**

**Pulmonary Function:** **p = 0.822**

**Conclusion**

This pilot Phase I-II Safety and tolerability study demonstrated acceptable safety profile of minocycline in combination with riluzole. Functional outcomes were also measured. No statistically significant differences were observed in secondary endpoints.

## NCT00047723 (Gordon et al., 2007)

**Compound:** Minocycline

**Sponsor:** NINDS

**Publication Year:**  2007

**Design:**  double-blind placebo controlled

**Phase:**  III

**Treatment duration:** 4 months lead-in + 9 months treatment

**Randomization:**  1:1

**Arms:**  Placebo

Minocycline escalating dose (up to a max of 400 mg/ twice daily)

**Patients/arm (total):** 206/206 (412)

**Primary endpoint:** ALSFRS-R

Alpha = 0.05

**Secondary endpoints:** FVC (forced vital capacity)

MMT (manual muscle testing)

Quality of Life

Survival

**Results**

**Primary endpoint: ALSFRS-R: Failure.** Faster progression was observed for patients in the minocycline (investigational drug) group (**p=0.005**)

**Secondary endpoints:** no significance on all endpoints, including survival and quality of life (see Table)


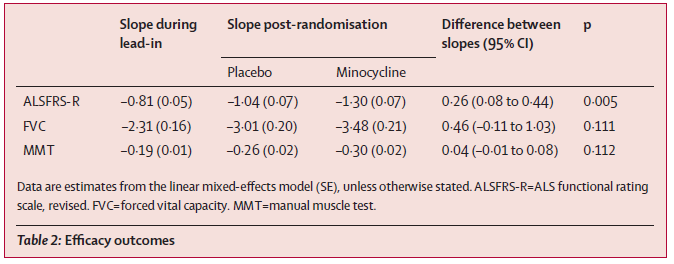


**Conclusion**

This large-scale Phase III trial was launched on the basis of the 3 earlier Phase I-II pilot studies which demonstrated acceptable safety and tolerability. Phase III trial described here failed on the primary endpoint and demonstrated statistically significant, treatment-related **worsening** of the symptoms based on the ALSFRS-R functional outcome. No statistical significance was reached in any of the secondary endpoints.

# NP001

## NCT01091142 (Miller et al., 2014)

**Compound:** NP001

**Sponsor:** Neuraltus Pharmaceuticals, Inc.

**Publication Year:**  2014

**Design:**  double-blind placebo controlled safety and tolerability

**Phase:**  I

**Treatment duration:** single dose (30 min infusion)

**Randomization:**  unspecified

**Arms:**  Placebo

NP001 (**0.2** // **0.8** // **1.6** // **3.2** mg/kg infusion)

**Patients/arm (total):** 8/6/6/6/6 (32)

**Primary endpoints:** Safety and tolerability

Changes in clinical status

**Secondary endpoints:** CD16 (blood monocyte immune activation marker)

HLA-DR blood monocyte immune activation marker)

**Results**

**Primary endpoints:**

**Safety and tolerability:** Deemed acceptable

**Clinical status:** Treatment did not adversely affect clinical status

**Secondary endpoints:**

**CD16**: NP001 treatment led to dose-dependent decreases in levels of HLA-DR. Best treatment effect was observed at the 1.6 mg/kg dose

**HLA-DR**: NP001 treatment led to decreased levels of HLA-DR (no dose dependency was noted)

**Conclusion**

This Phase I Study demonstrated that NP001 treatment is safe in ALS patients. NP001 appeared to have a general anti-inflammatory effect as measured by reversal of abnormal HLA-DR expression. Given that the best effect on CD16 marker was achieved on a 1.6 mg/kg dose, authors suggested that for Phase II follow-up study, 2 dose ranges could be used: 1.0 mg/kg (minimally effective dose) and 2.0 mg/kg (apparently immune regulatory dose). Phase II study was subsequently launched.

## NCT01281631 (Miller et al., 2015)

**Compound:** NP001

**Sponsor:** Neuraltus Pharmaceuticals, Inc.

**Publication Year:**  2015

**Design:**  exploratory double-blind placebo controlled

**Phase:**  II

**Treatment duration:** 6 months

**Randomization:**  1:1:1

**Arms:**  Placebo

NP001 (**1.0** // **2.0** mg/kg 30 min daily infusions over 6 cycles, with 4 weeks between each cycle)

**Patients/arm (total):** 42/49/45 (136)

**Primary endpoints:** ALSFRS-R (baseline to treatment end)

Alpha = 0.10

**Secondary endpoints:** ALSFRS-R (baseline to treatment end and follow-up)

SVC (slow vital capacity)

Survival

Time to tracheostomy

This trial was designed as an exploratory safety, tolerability, and preliminary efficacy study that was underpowered to detect even a large slowing of progression (>30%).

**Results**

**Primary endpoint:** not significant

**Secondary endpoints:** no significance on any endpoints

**Conclusion**

This exploratory Phase II trial failed on the primary endpoint. No statistical significance was reached in any of the secondary endpoints. However, halting of symptom progression in a dose dependent fashion in a subset of patients was observed: 25% vs 19% vs 11% in the 2 mg/kg, 1 mg/kg, and placebo groups, respectively. The clinical features of these responders were not different from nonresponders, therefore, authors conclude that it is unclear what baseline factors might allow identification of patients who would be more likely to respond to drug. Authors further conclude that if these factors could be elucidated, then it may be possible to launch an additional clinical trial with this compound in the future.

# Olesoxine

## NCT00868166 (Lenglet et al., 2014)

**Compound:** Olesoxine

**Sponsor:** Trophos/European Union

**Publication Year:**  2014

**Design:**  double-blind placebo controlled

**Phase:**  II-III

**Treatment duration:** 18 months

**Randomization:**  1:1

**Arms:**  Placebo + riluzole

Olesoxine 330 mg /day + riluzole

**Patients/arm (total):** 253/259 (512)

**Primary endpoint:** Survival

Alpha = 0.05

**Secondary endpoints:** Time to death **or** NIV (permanent non-invasive ventilation) **or** tracheostomy

ALSFRS-R

SVC (slow vital capacity)

MMT (manual muscle testing)

**Results**

**Primary endpoint:**

**Survival:** Estimated overall survival according to Kaplan–Meier analysis was 67.5% (95% CI 61.0%–73.1%) in the placebo group and 69.4% (95% CI 63.0%–74.9%) in the olesoxime group. **Not significant**

**Secondary endpoints:** no significance on any of the endpoints

**Conclusion**

This large-scale Phase III trial was launched on the basis of encouraging preclinical results. Prior Phase I safety trial had demonstrated acceptable safety profile with olesoxine alone, or in combination with riluzole in healthy volunteers.

Phase III study failed on the primary endpoint. No statistical significance was reached in any of the secondary endpoints.

# Pioglitazone

## NCT00690118 (Dupuis et al., 2012)

**Compound:** Pioglitazone

**Sponsor:** Takeda Pharma GmbH

**Publication Year:**  2012

**Design:**  double-blind placebo controlled

**Phase:**  II

**Treatment duration:** 4 week lead-in + 18 months treatment

**Randomization:**  1:1

**Arms:**  Placebo + riluzole

Pioglitazone 45 mg/day + riluzole

**Patients/arm (total):** 110/109 (219)

**Primary endpoint:** Survival

Alpha = 0.025

**Secondary endpoints** ALSFRS-R

SVC (slow vital capacity)

time to tracheotomy

Time to NIV (non-invasive ventilation)

EUROQoL EQ-5D (quality of life)

**Results**

**Primary endpoint:** not significant (**p=0.48**)

**Secondary endpoints:** no statistical significance in any of the endpoints

**Conclusion**

This Phase II study was launched on the basis of positive preclinical data. No prior Phase I-II Studies were performed. The study failed on the primary endpoint. No statistical significance was reached in any of the secondary endpoints.

# Talampanel

## Pascuzzi 2010 (Pascuzzi et al., 2010)

**Compound:** Talampanel

**Sponsor:** Teva Pharmaceutical Industries

**Publication Year:**  2009

**Design:**  double-blind placebo controlled

**Phase:**  II

**Treatment duration:** 9 months

**Randomization:**  1:2

**Arms:**  Placebo

Talampanel 50 mg / 3x day

**Patients/arm (total):** 19/39 (58)

**Primary endpoint:** TQNE (rate of decline in isometric arm strength)

Alpha = 0.05

**Secondary endpoints:** VC

Isometric leg strength (TQNE)

Fine motor function (TQNE)

ALSFRS

Survival

**Results**

**Primary endpoint:** not significant

**Secondary endpoints:** no significance on all endpoints (see Table)


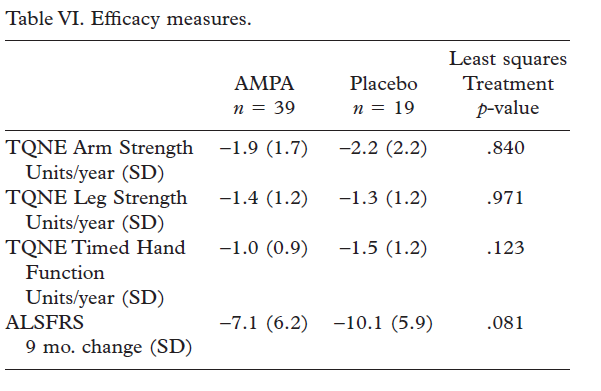


**Conclusion**

Phase II study failed on the primary endpoint. No statistical significance was reached in any of the secondary endpoints. Authors state that the study was completed in the 1990s, but for logistical reasons, results were not published until 2010. Authors also state that based on efficacy trends, a Phase III trial was underway. According to clinicaltrials.gov records, the Phase III trial was reclassified as a Phase II (NCT00696332). That study had subsequently failed, and the development for ALS was terminated in 2010.

## NCT00696332 (Teva, 2010)

**Compound:** Talampanel

**Sponsor:** Teva Pharmaceutical Industries

**Publication Year:**  2010

**Design:**  double-blind placebo controlled

**Phase:**  II

**Treatment duration:** 52 weeks

**Randomization:**  1:1:1

**Arms:**  Placebo

Talampanel 25 mg / 3x day

Talampanel 50 mg / 3x day

**Patients/arm (total):** ??/ ??/ ?? (559)

**Primary endpoint:** ALSFRS-R

Alpha = 0.05

**Secondary endpoints:** DTP (time to death **or** tracheotomy **or** PAV)

**Results**

**Primary endpoint:** not significant

**Secondary endpoints:** unknown

**Conclusion**

This large-scale Phase II study failed on the primary endpoint. Teva Pharmaceuticals never publicly released the results of this clinical trial. No additional information, beyond the press-release was ever presented(Teva, 2010). Drug development for ALS was terminated in 2010.

# TCH346

## NCT00072709 (Miller et al., 2007)

**Compound:** TCH346

**Sponsor:** Novartis

**Publication Year:**  2007

**Design:**  double-blind placebo controlled

**Phase:**  II-III

**Treatment duration:** 16 weeks lead-in + 24 weeks treatment

**Randomization:**  1:1:1:1:1

**Arms:**  Placebo

TCH346 **1.0** mg // **2.5** mg // **7.5** mg // **15** mg **per** day

**Patients/arm (total):** 111/108/113/112/109 (553)

**Primary endpoint:** ALSFRS-R

Alpha = 0.05

**Secondary endpoints:** TTD (time to death **or** tracheostomy **or** PAV)

FVC (forced vital capacity)

MMT (manual muscle testing)

This study used a novel design, whereby each patient’s individual decline during the 16-week lead-in period was compared to the decline observed during the treatment period.

**Results**

**Primary endpoint:** no significance at any dose.

**Secondary endpoint:** no significance on all endpoints

**Conclusion**

Phase II-III study failed on the primary endpoint. No statistical significance was reached in any of the secondary endpoints.

This large-scale Phase II-III clinical trial was launched on the basis of preclinical data and an earlier Phase II safety trial in 45 patients (NCT00036413 – results unpublished), which had demonstrated acceptable safety and had shown trends for efficacy.

# Tirasemtiv

## NCT01089010 (Shefner et al., 2012)

**Compound:** Tirasemtiv

**Sponsor:** Cytokinetics

**Publication Year:**  2012

**Design:**  placebo-controlled, three-period cross-over*

**Phase:**  II

**Treatment duration:** single dose administrations

**Randomization:**  not applicable (all patients received placebo, 250 mg and 500 mg tirasemtiv)

**Arms:**  Placebo

Tirasemtiv 250 mg

Tirasemtiv 500 mg

**Patients/arm (total):** 67

**Primary endpoint:** no pre-specified primary endpoint

**Secondary endpoints:** SVC (slow vital capacity)

SNIP (sniff nasal inspiratory pressure)

MVV (maximum voluntary ventilation)

HHD (hand-held dynamometry – muscle strength)

Global assessment (« do you feel the same, better or worse ? »)

Ashworth spasticity

Grip strength

*The purpose of the study was to assess safety and tolerability of tirasemtiv in ALS patients. In addition, assessment of the Evidence of Effect (EoE) using a wide-range of functional outcome measures was performed in order to determine the parameters to be tested in the follow-up studies.

**Results**

Safety and tolerability deemed acceptable. No pre-specified **p** value for functional outcomes was designated. Nominal p values are presented with an alpha of **0.05**.

**Global and functional assessments:** Both patients ’ and investigators ’ global assessments revealed a dose dependent increase in the proportion of patients perceived as better at 6 h after tirasemtiv (**p=0.071** and **p=0.015**, respectively, for the dose response). After 24 h after dosing, this effect was no longer evident.

**Endurance:** In the weaker hand, trends (**p < 0.10** and **> 0.05**) towards improvement in time taken for grip strength to fall to 70% or 60% of target for the 500-mg dose were noted at 6 h postdose; a similar pattern was also seen in the stronger hand.

**Respiratory measures**: No statistically significant changes in **SNIP** and **SVC**; however, performance improved for **MVV**, which tests both inspiration and expiration over a sustained time-period, and is thus susceptible to fatigue. The change was greatest at 24 h after the 500-mg dose (**p = 0.016**, δ = 4.17 l/min, 95% CI 0.80 - 7.54)

**Muscle strength:** not significant

**Conclusion:** None of the functional outcomes can be considered as proof of efficacy. Reported p values simply indicate that the drug may be efficacious, as all the measures were taken after **a single dose administration**. This study was used **a guideline for the design of follow-up studies.**

## NCT01378676 (Parts 1 and 2) (Shefner et al., 2013a)

**Compound:** Tirasemtiv

**Sponsor:** Cytokinetics

**Publication Year:**  2013

**Design:**  double-blind placebo controlled exploratory*

**Phase:**  II

**Treatment duration:** 7 days lead-in + 2 weeks treatment

**Randomization:**  1:1:1:1

**Arms:**  Placebo

Tirasemtiv **125** mg // **250** mg // **375** mg **per** day

**Patients/arm (total):** 13/12/12/12 (49)

**Primary endpoint:** Safety and tolerability

**Secondary endpoints:** ALSFRS-R

SVC (slow vital capacity)

SNIP (sniff nasal inspiratory pressure)

MVV (maximum voluntary ventilation)

HHD (hand-held dynamometry – muscle strength)

Grip strength

*The study was divided into 2 parts with identical design, except that:

- In Part 1, patients were not on riluzole (a total of 24 patients were randomized into 4-treatment arms)
- In Part 2, all the patients were on reduced dose of riluzole (a total of 25 patients in 4 arms)

For the purposes of efficacy analyses of functional outcomes, all the patients from Parts 1 and 2 of the study were pooled together.

**Results**

**Primary endpoint:** Acceptable safety and tolerability

**Secondary endpoints:**

**ALSFRS-R:** due to the small sample size, ALSFRS-R values obtained on D8 and D15 were pooled together (**p=0.10**)

**SVC, SNIP, MVV**: not significant

**HHD:** not significant

**Grip strength:** no improvement (**slope, -6.5;** **p=0.0777**)

**Conclusion**

This was a 3-part exploratory Phase II study designed to measure safety and tolerability of tirasemtiv in ALS patients with and without concomitant riluzole use. No statistical significance was reached in any of the efficacy endpoints.

## NCT01378676 (Part 3) (Shefner et al., 2013a)

**Compound:** Tirasemtiv

**Sponsor:** Cytokinetics

**Publication Year:**  2013

**Design:**  ascending dose double blind placebo controlled exploratory

**Phase:**  II

**Treatment duration:** 7 days lead-in + 3 weeks treatment

**Randomization:**  unspecified

**Arms:**  Placebo

Tirasemtiv weekly dose escalation (from **250** to **375** to **500** mg /day)

**Patients/arm (total):** 6/21 (27)

**Primary endpoint:** Safety and tolerability

**Secondary endpoints:** ALSFRS-R

SVC (slow vital capacity)

SNIP (sniff nasal inspiratory pressure)

MVV (maximum voluntary ventilation)

HHD (hand-held dynamometry – muscle strength)

Grip strength

**Results**

**Primary endpoint:** Acceptable safety and tolerability

**Secondary endpoints:**

**ALSFRS-R:** Because of dose escalation, dose dependency was not calculated. At day22, the LS Mean ALSFRS-R change from baseline was **1.6** for the active treatment group and **1.9** for the placebo group (**no p reported**).

**SVC, SNIP, MVV**: not significant

**HHD:** not significant

**Grip strength:** trend to improvement (**slope, 9.8; p=0.0777**)

**Conclusion**

This was a 3-part exploratory Phase II study designed to measure safety and tolerability of tirasemtiv in ALS patients with and without concomitant riluzole use. No statistical significance was reached in any of the efficacy endpoints.

Phase II meta-analysis (Shefner et al., 2013b)

**Compound:** Tirasemtiv

**Sponsor:** Cytokinetics

**Publication Year:**  2013

**Design:**  meta-analysis of 3 Phase II studies presented in the preceding section

**Phase:**  II

**Treatment duration:** single dose // 2 weeks // 3 weeks

**Randomization:**  not applicable

**Arms:**  Placebo

Tirasemtiv **125** mg // **250** mg // **375** mg// **500** mg **per** day

**Patients/arm (total):** 143

**Primary analysis:** Relationship between functional outcomes and serum tirasemtiv levels

**Functional outcomes:** ALSFRS-R

SVC (slow vital capacity)

SNIP (sniff nasal inspiratory pressure)

MVV (maximum voluntary ventilation)

HHD (hand-held dynamometry – muscle strength – Mega Score)

Grip strength

**Results**

The tirasemtiv plasma concentrations in these studies ranged from undetectable on placebo to approximately 15 μg/ml.

**ALSFRS-R:** a trend toward higher ALSFRS-R scores as a function of the average tirasemtiv concentration was noted (0.11 points/μg/ml; **p= 0.089**), however, only about half of the patients included in this post hoc analysis (76/143) underwent this evaluation.

**SVC:** SVC was not significantly correlated with tirasemtiv concentration (**p=0.689**)

**SNIP:** increases from baseline in SNIP were statistically significantly related to increases in the tirasemtiv concentration (0.47 cm H2O/μg/ml, **p=0.0036**)

**MVV**: increases from baseline in MVV were statistically significantly related to increases in the tirasemtiv concentration (0.33 l/minute/μg/ml, **p=0.022**)

**HHD:** The isometric strength megascore changes from baseline were statistically significantly related to increases in the tirasemtiv concentration (**p=0.002**)

**Grip strength:** There was a trend toward increased handgrip endurance as a function of tirasemtiv concentration in the stronger hand (0.12 s/ μ g/ml, **p=0.078**); a similar trend was noted for the weaker hand (0.63 s/ μ g/ml, **p=0.039**)

**Conclusion**

Meta-analysis presented here provided encouragement for the continued evaluation of tirasemtiv in ALS patients. A large-scale Phase IIb study was subsequently launched.

## NCT01709149 (Shefner et al., 2016)

**Compound:** Tirasemtiv

**Sponsor:** Cytokinetics

**Publication Year:**  2016

**Design:**  double-blind placebo controlled

**Phase:**  IIb

**Treatment duration:** 1 week open-label + 12 weeks randomized

**Randomization:**  1:1

**Arms:**  Placebo

Tirasemtiv (**1 week:** 125mg/b.i.d.; + **1 week**: 125 mg in the morning and 250 mg at night; **followed** by 250 mg/b.i.d. till end of treatment)

**Patients/arm (total):** 302/303 (605)

**Primary endpoint:** ALSFRS-R (change from baseline to average scores at weeks **8** and **12**)

Alpha = 0.05

**Secondary endpoints:** SVC (slow vital capacity)

MVV (maximum voluntary ventilation)

SNIP (sniff nasal inspiratory pressure)

HHD (hand-held dynamometry – muscle strength – Mega-Score)

Grip strength (handgrip fatigue)

**Results**

**Primary endpoint: ALSFRS-R:** Failure (**p=0.1572**); see figure

**Secondary endpoints:** Statistical significance on **SVC** and **HHD**; see figure


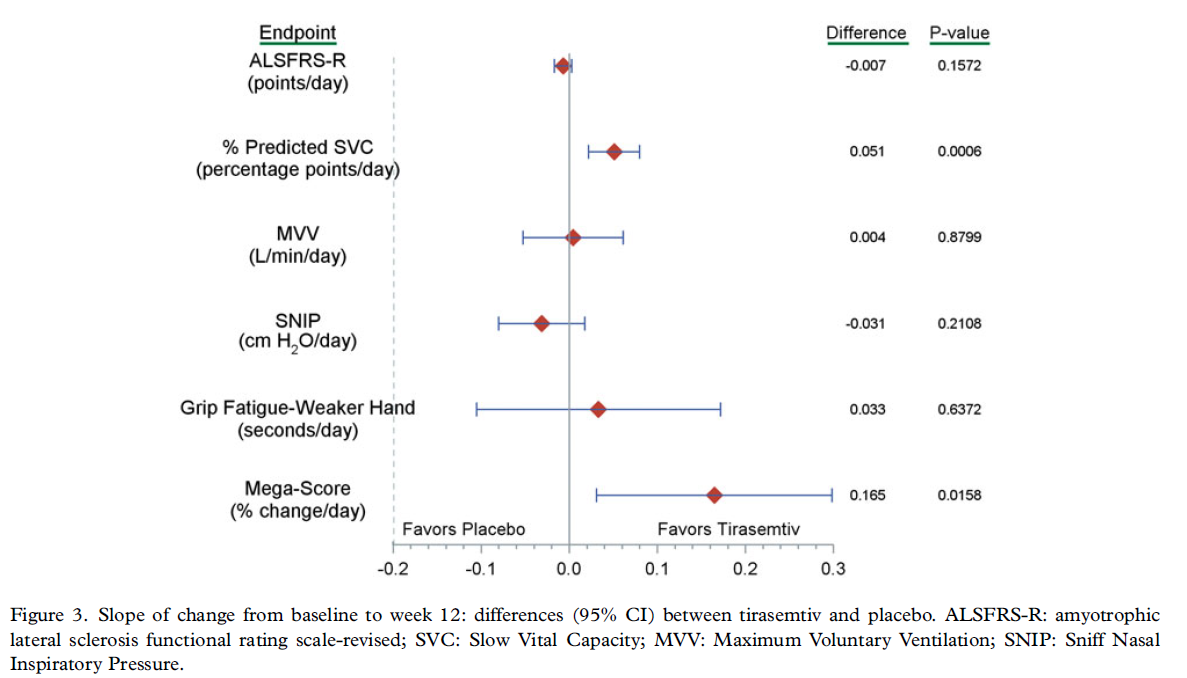


**Conclusion**

This large-scale Phase IIb study failed on the primary endpoint. Statistical significance was reached for 2 of the secondary endpoints (slow vital capacity and muscle strength). Authors cite tirasemtiv tolerability issues as one of the reasons for study failure. Authors consider these results sufficient to justify the launch of an additional Phase III study.

# Valproic acid

## NCT00136110 (Piepers et al., 2009)

**Compound:** Valproic acid + riluzole

**Sponsor:** Prinses Beatrix Fonds (Netherlands)

**Publication Year:**  2009

**Design:**  sequential, double-blind placebo controlled

**Phase:**  III

**Treatment duration:** 12 months

**Randomization:**  1:1

**Arms:**  Placebo

Valproic acid 1500 mg/day

**Patients/arm (total):** 81/82 (163)

**Primary endpoint:** DTP (time to death **or** tracheostomy **or** PAV)

Alpha = 0.05

**Secondary endpoints** ALSFRS-R

**Results**

**Primary endpoint:** not significant

**Secondary endpoint:** not significant

**Conclusion:**

This large-scale Phase III clinical trial was launched on the basis of positive preclinical data and had employed sequential design, which allows inclusion of more patients if the treatment effect was observed. The study failed on the primary endpoint. No statistical significance was reached in a secondary endpoint.

# Xaliproden

## Lacomblez – PII (Lacomblez et al., 2004)

**Compound:** Xaliproden

**Sponsor:** Sanofi - Synthelabo

**Publication Year:**  2004

**Design:** Multipart placebo-controlled safety, **safety-efficacy**, PK, high-dose tolerability and open-label long-term safety studies

**Phase:**  II

**Treatment duration:** 32 weeks

**Randomization:**  1:1:1:1

**Arms:**  Placebo

Xaliproden 0.5, 1.0, 2.0 mg/day

**Patients/arm (total):** 27/8/9/27 (70)

**Efficacy Evaluation:** conducted in 2.0 mg /day Xaliproden versus placebo arms only

**Primary endpoint:** MMT (22 muscles) *manual muscle test

Alpha = 0.05

**Secondary endpoints:** BFS (bulbar function, assessed with Norris scale))

FVC% (forced vital capacity)

LFS (limb function)

**Results**

**Primary endpoint: P < 0.10**

**Secondary endpoints:**

**BFS**: **P < 0.10**

**FVC%: P < 0.10**

**LFS: P < 0.10**

**Conclusion**

Phase II study failed on the primary endpoint. No statistical significance was reached in any of the secondary endpoints.

**Post-hoc analysis on the completer population**

**MMT: P=0.08**

**BFS**: not significant

**FVC%: P=0.046**

**LFS: P=0.09**

## EFC2941 (Meininger et al., 2004)

**Compound:** Xaliproden

**Sponsor:** Sanofi - Synthelabo

**Publication Year:**  2004

**Design:** double-blind placebo controlled

**Phase:**  III

**Treatment duration:** 18 months

**Randomization:**  1:1:1

**Arms:**  Placebo

Xaliproden 1.0, 2.0 mg/day

**Patients/arm (total):** 286/293/288 (867)

**Primary endpoints:** time to VC <50% or DTP*****

Alpha = 0.0125, adjusted for 2-dose group comparisons with placebo and 2 primary endpoints (a=0.05 divided by 4)

**Secondary endpoints:** ALSFRS

CGI (Clinical Global Impression)

Manual muscle testing

Norris scale

SIP (sickness impact profile)

Spasticity scale

VAS (Visual analogue scales)

VC (Vital capacity)

**^$^**DTP (Time to death **or** tracheostomy **or** PAV)

***** The calculation of this endpoint was changed at the recommendation of the FDA before unblinding. Initially planned primary analysis was time to VC <50%, without taking into consideration that death, tracheostomy or PAV may occur before VC <50% is reached.

**Results**

**Primary endpoints:** For the primary endpoints, a comparison of Kaplan-Meier survival curves of each xaliproden group with the placebo group was made using a 2-sided, stratified (bulbar vs. limb site of onset) log-rank test (unadjusted RRR). In a secondary analysis (adjusted RRR), for each primary endpoint, the Cox proportional hazards model analysis was performed to adjust for possible imbalance in prognostic factors. Both analyses were predefined.

On the basis of the FDA-revised primary endpoint, the study failed with the two doses.


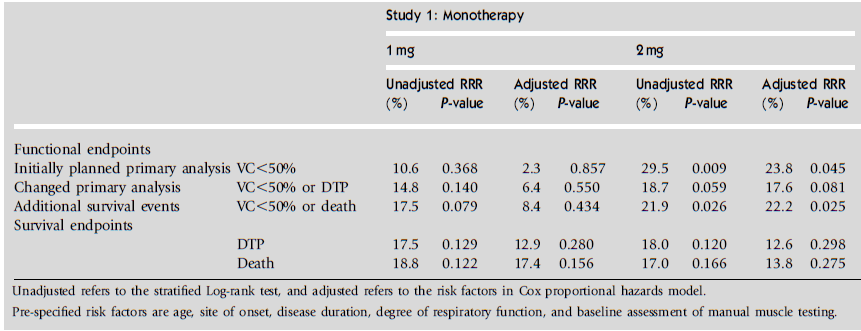


**Secondary endpoints:**

**ALSFRS:** slopes **1 mg**: 19% reduction (**P=0.013**); **2 mg**: 13% reduction (**P<0.10**)

**CGI (Clinical Global Impression): 1 mg**: not significant; **2 mg**: not significant

**Manual muscle testing: 1 mg**: not significant; **2 mg**: not significant

**Norris scale: 1 mg**: not significant; **2 mg**: not significant

**SIP (sickness impact profile): 1 mg**: not significant; **2 mg**: not significant

**Spasticity scale: 1 mg**: not significant; **2 mg**: not significant

**VAS (Visual analogue scales): 1 mg**: not significant; **2 mg**: not significant

**VC (Vital capacity):** slopes **1 mg**: 15% reduction (**P<0.10**); **2 mg**: 14% reduction (**P<0.10**)

**Conclusion**

Phase III study failed on both primary endpoints. No statistical significance was reached in any of the secondary endpoints, apart from ALSFRS slope decline, with statistical significance reached in 1 mg Xaliproden dose group only.

## EFC1923 (Meininger et al., 2004)

**Compound:** Xaliproden + Riluzole

**Sponsor:** Sanofi - Synthelabo

**Publication Year:**  2004

**Design:** double-blind placebo controlled

**Phase:**  III

**Treatment duration:** 18 months

**Randomization:**  1:1:1

**Arms:**  Placebo

Xaliproden 1.0, 2.0 mg/day

**Patients/arm (total):** 406/394/410 (1210)

**Primary endpoints:** time to VC <50% or DTP*****

Alpha = 0.0125, adjusted for 2-dose group comparisons with placebo and 2

primary endpoints (a=0.05 divided by 4)

**Secondary endpoints:** ALSFRS

CGI (Clinical Global Impression)

Manual muscle testing

Norris scale

SIP (sickness impact profile)

Spasticity scale

VAS (Visual analogue scales)

VC (Vital capacity)

**^$^**DTP (Time to death **or** tracheostomy **or** PAV)

***** The calculation of this endpoint was changed at the recommendation of the FDA before unblinding. Initially planned primary analysis was time to VC <50%, without taking into consideration that death, tracheostomy or PAV may occur before VC <50% is reached.

**Results**

**Primary endpoints:** For the primary endpoints, a comparison of Kaplan-Meier survival curves of each xaliproden group with the placebo group was made using a 2-sided, stratified (bulbar vs. limb site of onset) log-rank test (unadjusted RRR). In a secondary analysis (Adjusted RRR), for each primary endpoint, the Cox proportional hazards model analysis was performed to adjust for possible imbalance in prognostic factors. Both analyses were predefined.

On the basis of the FDA-revised primary endpoint, the study failed with the two doses.


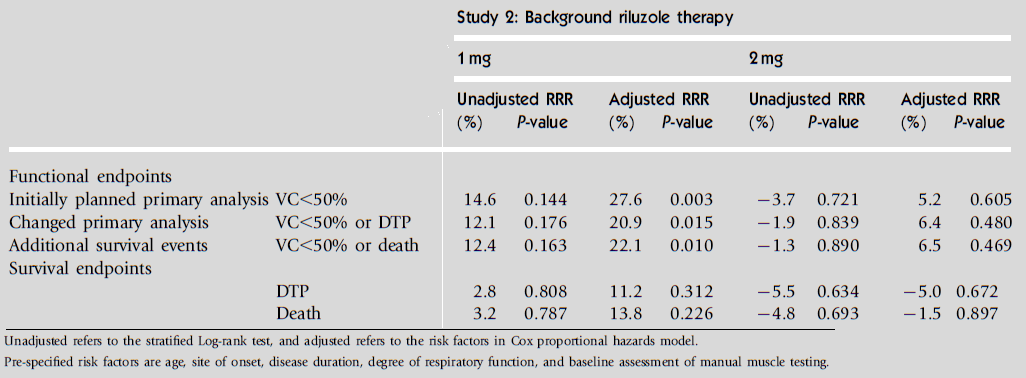


**Secondary endpoints:**

**ALSFRS:** slopes **1 mg**: not significant; **2 mg**: not significant

**CGI (Clinical Global Impression): 1 mg**: not significant; **2 mg**: not significant

**Manual muscle testing: 1 mg**: not significant; **2 mg**: not significant

**Norris scale: 1 mg**: not significant; **2 mg**: not significant

**SIP (sickness impact profile): 1 mg**: not significant; **2 mg**: not significant

**Spasticity scale: 1 mg**: not significant; **2 mg**: not significant

**VAS (Visual analogue scales): 1 mg**: not significant; **2 mg**: not significant

**VC (Vital capacity):** slopes **1 mg**: not significant; **2 mg**: not significant

**Conclusion**

Phase III study failed on both primary endpoints. No statistical significance was reached in any of the secondary endpoints.

# Bibliography

Abe, K., Itoyama, Y., Sobue, G., Tsuji, S., Aoki, M., Doyu, M., et al. (2014). Confirmatory double-blind, parallel-group, placebo-controlled study of efficacy and safety of edaravone (MCI-186) in amyotrophic lateral sclerosis patients. *Amyotroph. Lateral Scler. Frontotemporal Degener.* 15, 610–7. doi:10.3109/21678421.2014.959024.

Aggarwal, S. P., Zinman, L., Simpson, E., McKinley, J., Jackson, K. E., Pinto, H., et al. (2010). Safety and efficacy of lithium in combination with riluzole for treatment of amyotrophic lateral sclerosis: a randomised, double-blind, placebo-controlled trial. *Lancet Neurol.* 9, 481–488. doi:10.1016/S1474-4422(10)70068-5.

ALS CNTF Treatment Study Group (1995). A phase I study of recombinant human ciliary neurotrophic factor (rHCNTF) in patients with amyotrophic lateral sclerosis. The ALS CNTF Treatment Study (ACTS) Phase I-II Study Group. *Clin. Neuropharmacol.* 18, 515–32. Available at: http://www.ncbi.nlm.nih.gov/pubmed/8681312 [Accessed June 8, 2016].

ALS CNTF Treatment Study Group (1996). A double-blind placebo-controlled clinical trial of subcutaneous recombinant human ciliary neurotrophic factor (rHCNTF) in amyotrophic lateral sclerosis. *Neurology* 46, 1244–1244. doi:10.1212/WNL.46.5.1244.

Berry, J. D., Shefner, J. M., Conwit, R., Schoenfeld, D., Keroack, M., Felsenstein, D., et al. (2013). Design and initial results of a multi-phase randomized trial of ceftriaxone in amyotrophic lateral sclerosis. *PLoS One* 8, e61177. doi:10.1371/journal.pone.0061177.

Borasio, G. D., Robberecht, W., Leigh, P. N., Emile, J., Guiloff, R. J., Jerusalem, F., et al. (1998). A placebo-controlled trial of insulin-like growth factor-I in amyotrophic lateral sclerosis. European ALS/IGF-I Study Group. *Neurology* 51, 583–6. Available at: http://www.ncbi.nlm.nih.gov/pubmed/9710040 [Accessed June 7, 2016].

Bozik, M. E., Mitsumoto, H., Brooks, B. R., Rudnicki, S. A., Moore, D. H., Zhang, B., et al. (2014). A post hoc analysis of subgroup outcomes and creatinine in the phase III clinical trial (EMPOWER) of dexpramipexole in ALS. *Amyotroph. Lateral Scler. Front. Degener.* 15, 406–413. doi:10.3109/21678421.2014.943672.

Bradley, W. G. (1995). A phase I/II study of recombinant brain-derived neurotrophic in patients with ALS. in *Annals of neurology*, 38:971.

de Carvalho, M., Pinto, S., Costa, J., Evangelista, T., Ohana, B., and Pinto, A. (2010). A randomized, placebo-controlled trial of memantine for functional disability in amyotrophic lateral sclerosis. *Amyotroph. Lateral Scler.* 11, 456–60. doi:10.3109/17482968.2010.498521.

Cudkowicz, M., Bozik, M. E., Ingersoll, E. W., Miller, R., Mitsumoto, H., Shefner, J., et al. (2011). The effects of dexpramipexole (KNS-760704) in individuals with amyotrophic lateral sclerosis. *Nat. Med.* 17, 1652–1656. doi:10.1038/nm.2579.

Cudkowicz, M. E., van den Berg, L. H., Shefner, J. M., Mitsumoto, H., Mora, J. S., Ludolph, A., et al. (2013a). Dexpramipexole versus placebo for patients with amyotrophic lateral sclerosis (EMPOWER): a randomised, double-blind, phase 3 trial. *Lancet. Neurol.* 12, 1059–67. doi:10.1016/S1474-4422(13)70221-7.

Cudkowicz, M. E., Shefner, J. M., Schoenfeld, D. A., Zhang, H., Andreasson, K. I., Rothstein, J. D., et al. (2006). Trial of celecoxib in amyotrophic lateral sclerosis. *Ann. Neurol.* 60, 22–31. doi:10.1002/ana.20903.

Cudkowicz, M., Shefner, J., and Consortium, N. (2013b). STAGE 3 Clinical Trial of Ceftriaxone in Subjects with ALS (S36.001). *Neurology* 80, S36.001. Available at: http://www.neurology.org/content/80/7_Supplement/S36.001.abstract [Accessed June 6, 2016].

Dupuis, L., Dengler, R., Heneka, M. T., Meyer, T., Zierz, S., Kassubek, J., et al. (2012). A randomized, double blind, placebo-controlled trial of pioglitazone in combination with riluzole in amyotrophic lateral sclerosis. *PLoS One* 7, e37885. doi:10.1371/journal.pone.0037885.

Fornai, F., Longone, P., Cafaro, L., Kastsiuchenka, O., Ferrucci, M., Manca, M. L., et al. (2008). Lithium delays progression of amyotrophic lateral sclerosis. *Proc. Natl. Acad. Sci. U. S. A.* 105, 2052–7. doi:10.1073/pnas.0708022105.

Gordon, P. H., Doorish, C., Montes, J., Mosley, R. L., Mosely, R. L., Diamond, B., et al. (2006). Randomized controlled phase II trial of glatiramer acetate in ALS. *Neurology* 66, 1117–9. doi:10.1212/01.wnl.0000204235.81272.e2.

Gordon, P. H., Moore, D. H., Gelinas, D. F., Qualls, C., Meister, M. E., Werner, J., et al. (2004). Placebo-controlled phase I/II studies of minocycline in amyotrophic lateral sclerosis. *Neurology* 62, 1845–7. Available at: http://www.ncbi.nlm.nih.gov/pubmed/15159491 [Accessed June 3, 2016].

Gordon, P., Moore, D., Miller, R., Florence, J., Verheijde, J., Doorish, C., et al. (2007). Efficacy of minocycline in patients with amyotrophic lateral sclerosis: a phase III randomised trial. *Lancet Neurol.* 6, 1045–1053. Available at: citeulike-article-id:3746644\nhttp://dx.doi.org/10.1016/S1474-4422(07)70270-3.

Groeneveld, G. J., Veldink, J. H., van der Tweel, I., Kalmijn, S., Beijer, C., de Visser, M., et al. (2003). A randomized sequential trial of creatine in amyotrophic lateral sclerosis. *Ann. Neurol.* 53, 437–45. doi:10.1002/ana.10554.

Kasarskis, E. J., Shefner, J. M., and Miller, R. (1999). A controlled trial of recombinant methionyl human BDNF in ALS: The BDNF Study Group (Phase III). *Neurology* 52, 1427–1433. doi:10.1212/WNL.52.7.1427.

Kaufmann, P., Thompson, J. L. P., Levy, G., Buchsbaum, R., Shefner, J., Krivickas, L. S., et al. (2009). Phase II trial of CoQ10 for ALS finds insufficient evidence to justify phase III. *Ann. Neurol.* 66, 235–44. doi:10.1002/ana.21743.

Lacomblez, L., Bensimon, G., Douillet, P., Doppler, V., Salachas, F., and Meininger, V. (2004). Xaliproden in amyotrophic lateral sclerosis: early clinical trials. *Amyotroph. Lateral Scler. Other Motor Neuron Disord.* 5, 99–106. doi:10.1080/14660820410018973.

Lai, E. C., Felice, K. J., Festoff, B. W., Gawel, M. J., Gelinas, D. F., Kratz, R., et al. (1997). Effect of recombinant human insulin-like growth factor-I on progression of ALS. A placebo-controlled study. The North America ALS/IGF-I Study Group. *Neurology* 49, 1621–1630. Available at: http://www.ncbi.nlm.nih.gov/pubmed/9409357 [Accessed June 7, 2016].

Lauria, G., Campanella, A., Filippini, G., Martini, A., Penza, P., Maggi, L., et al. (2009). Erythropoietin in amyotrophic lateral sclerosis: a pilot, randomized, double-blind, placebo-controlled study of safety and tolerability. *Amyotroph. Lateral Scler.* 10, 410–5. doi:10.3109/17482960902995246.

Lauria, G., Dalla Bella, E., Antonini, G., Borghero, G., Capasso, M., Caponnetto, C., et al. (2015). Erythropoietin in amyotrophic lateral sclerosis: a multicentre, randomised, double blind, placebo controlled, phase III study. *J. Neurol. Neurosurg. Psychiatry* 86, 879–86. doi:10.1136/jnnp-2014-308996.

Lenglet, T., Lacomblez, L., Abitbol, J. L., Ludolph, A., Mora, J. S., Robberecht, W., et al. (2014). A phase II-III trial of olesoxime in subjects with amyotrophic lateral sclerosis. *Eur. J. Neurol.* 21, 529–536. doi:10.1111/ene.12344.

Meininger, V., Bensimon, G., Bradley, W. R., Brooks, B., Douillet, P., Eisen, A. a, et al. (2004). Efficacy and safety of xaliproden in amyotrophic lateral sclerosis: results of two phase III trials. *Amyotroph. Lateral Scler. Other Motor Neuron Disord.* 5, 107–117. doi:10.1080/14660820410019602.

Meininger, V., Drory, V. E., Leigh, P. N., Ludolph, A., Robberecht, W., and Silani, V. (2009). Glatiramer acetate has no impact on disease progression in ALS at 40 mg/day: a double- blind, randomized, multicentre, placebo-controlled trial. *Amyotroph. Lateral Scler.* 10, 378–83. doi:10.3109/17482960902803432.

Miller, R., Bradley, W., Cudkowicz, M., Hubble, J., Meininger, V., Mitsumoto, H., et al. (2007). Phase II/III randomized trial of TCH346 in patients with ALS. *Neurology* 69, 776–84. doi:10.1212/01.wnl.0000269676.07319.09.

Miller, R., Bryan, W., and Munsat, T. (1993). Safety, tolerability and pharmacokinetics of recombinant human ciliary neurotrophic factor (rhCNTF) in patients with amyotrophic lateral sclerosis (ALS). in *Annals of neurology*, 34:241.

Miller, R. G., Block, G., Katz, J. S., Barohn, R. J., Gopalakrishnan, V., Cudkowicz, M., et al. (2015). Randomized phase 2 trial of NP001-a novel immune regulator: Safety and early efficacy in ALS. *Neurol. Neuroimmunol. Neuroinflammation* 2, e100–e100. doi:10.1212/NXI.0000000000000100.

Miller, R. G., Petajan, J. H., Bryan, W. W., Armon, C., Barohn, R. J., Goodpasture, J. C., et al. (1996). A placebo-controlled trial of recombinant human ciliary neurotrophic (rhCNTF) factor in amyotrophic lateral sclerosis. rhCNTF ALS Study Group. *Ann. Neurol.* 39, 256–260. doi:10.1002/ana.410390215.

Miller, R. G., Zhang, R., Block, G., Katz, J., Barohn, R., Kasarskis, E., et al. (2014). NP001 regulation of macrophage activation markers in ALS: A phase I clinical and biomarker study. *Amyotroph. Lateral Scler. Front. Degener.* 15, 601–609. doi:10.3109/21678421.2014.951940.

Morrison, K. E., Dhariwal, S., Hornabrook, R., Savage, L., Burn, D. J., Khoo, T. K., et al. (2013). Lithium in patients with amyotrophic lateral sclerosis (LiCALS): a phase 3 multicentre, randomised, double-blind, placebo-controlled trial. *Lancet. Neurol.* 12, 339–45. doi:10.1016/S1474-4422(13)70037-1.

Pascuzzi, R. M., Shefner, J., Chappell, A. S., Bjerke, J. S., Tamura, R., Chaudhry, V., et al. (2010). A phase II trial of talampanel in subjects with amyotrophic lateral sclerosis. *Amyotroph. Lateral Scler.* 11, 266–71. doi:10.3109/17482960903307805.

Pastula, D. M., Moore, D. H., and Bedlack, R. S. (2012). Creatine for amyotrophic lateral sclerosis/motor neuron disease. *Cochrane database Syst. Rev.* 12, CD005225. doi:10.1002/14651858.CD005225.pub3.

Piepers, S., Veldink, J. H., De Jong, S. W., Van Der Tweel, I., Van Der Pol, W. L., Uijtendaal, E. V, et al. (2009). Randomized sequential trial of valproic acid in amyotrophic lateral sclerosis. *Ann. Neurol.* 66, 227–234. doi:10.1002/ana.21620.

Pontieri, F. E., Ricci, A., Pellicano, C., Benincasa, D., and Buttarelli, F. R. (2005). Minocycline in amyotrophic lateral sclerosis: a pilot study. *Neurol. Sci.* 26, 285–7. doi:10.1007/s10072-005-0474-x.

Rosenfeld, J. (2001). Creatine monohydrate in amyotrophic lateral sclerosis: preliminary results. in *American Academy of Neurology Annual Meeting*.

Rosenfeld, J., King, R. M., Jackson, C. E., Bedlack, R. S., Barohn, R. J., Dick, A., et al. (2008). Creatine monohydrate in ALS: effects on strength, fatigue, respiratory status and ALSFRS. *Amyotroph. Lateral Scler.* 9, 266–72. doi:10.1080/17482960802028890.

Shefner, J., Cedarbaum, J. M., Cudkowicz, M. E., Maragakis, N., Lee, J., Jones, D., et al. (2012). Safety, tolerability and pharmacodynamics of a skeletal muscle activator in amyotrophic lateral sclerosis. *Amyotroph. Lateral Scler.* 13, 430–438. doi:10.3109/17482968.2012.684214.

Shefner, J. M., Cudkowicz, M. E., Schoenfeld, D., Conrad, T., Taft, J., Chilton, M., et al. (2004). A clinical trial of creatine in ALS. *Neurology* 63, 1656–61. Available at: http://www.ncbi.nlm.nih.gov/pubmed/15534251 [Accessed May 27, 2016].

Shefner, J. M., Watson, M. Lou, Meng, L., and Wolff, A. A. (2013a). A study to evaluate safety and tolerability of repeated doses of tirasemtiv in patients with amyotrophic lateral sclerosis. *Amyotroph. Lateral Scler. Frontotemporal Degener.* 14, 574–581. doi:10.3109/21678421.2013.822517.

Shefner, J. M., Wolff, A. A., and Meng, L. (2013b). The relationship between tirasemtiv serum concentration and functional outcomes in patients with ALS. *Amyotroph Lateral Scler Front. Degener* 14, 582–585. doi:10.3109/21678421.2013.817587.

Shefner, J. M., Wolff, A. A., Meng, L., Bian, A., Lee, J., Barragan, D., et al. (2016). A randomized, placebo-controlled, double-blind phase IIb trial evaluating the safety and efficacy of tirasemtiv in patients with amyotrophic lateral sclerosis. *Amyotroph. Lateral Scler. Frontotemporal Degener.*, 1–10. doi:10.3109/21678421.2016.1148169.

Sorenson, E. J., Windbank, A. J., Mandrekar, J. N., Bamlet, W. R., Appel, S. H., Armon, C., et al. (2008). Subcutaneous IGF-1 is not beneficial in 2-year ALS trial. *Neurology* 71, 1770–1775. doi:10.1212/01.wnl.0000335970.78664.36.

Tanaka, M., Sakata, T., Palumbo, J., and Akimoto, M. (2016a). A 24-Week, Phase III, Double-Blind, Parallel-Group Study of Edaravone (MCI-186) for Treatment of Amyotrophic Lateral Sclerosis (ALS) (P3.189). *Neurology* 86, P3.189.

Tanaka, M., Sakata, T., Palumbo, J., and Akimoto, M. (2016b). A Double-Blind, Parallel-Group, Placebo-Controlled, 24-Week, Exploratory Study of Edaravone (MCI-186) for the Treatment of Advanced Amyotrophic Lateral Sclerosis (ALS) (P3.191). *Neurology* 86, P3.191.

Teva (2010). News Release. *Teva Pharm.* Available at: http://ir.tevapharm.com/phoenix.zhtml?c=73925&p=irol-newsArticle&ID=1555496.

Verstraete, E., Veldink, J. H., Huisman, M. H. B., Draak, T., Uijtendaal, E. V, van der Kooi, A. J., et al. (2012). Lithium lacks effect on survival in amyotrophic lateral sclerosis: a phase IIb randomised sequential trial. *J. Neurol. Neurosurg. Psychiatry* 83, 557–64. doi:10.1136/jnnp-2011-302021.

Yoshino, H., and Kimura, A. (2006). Investigation of the therapeutic effects of edaravone, a free radical scavenger, on amyotrophic lateral sclerosis (Phase II study). *Amyotroph. Lateral Scler.* 7, 241–5. doi:10.1080/17482960600881870.
